# Surrogate-based optimization design for surface texture of helical pair in helical hydraulic rotary actuator

**DOI:** 10.1038/s41598-023-47509-7

**Published:** 2023-11-20

**Authors:** Song Liu, Baoren Li, Runlin Gan, Yue Xu, Gang yang

**Affiliations:** FESTO Pneumatics Centre, School of Mechanical Science & Engineering, Huazhong University of Science and Technology, Wuhan, 430074 China

**Keywords:** Engineering, Mechanical engineering

## Abstract

A good surface texture design can effectively improve the tribological performance of the helical pair within a helical hydraulic rotary actuator(HHRA). However, the optimization design process can be time-consuming due to the multiple design variables involved and the complexity of the mathematical model. This paper proposes a modified efficient global optimization (MEGO) method for solving such demanding surface texture design challenges. The MEGO utilizes a Kriging model with the optimized Latin hypercube sampling (OLHS) for initial sampling and the proposed modified expected improvement (MEI) function for sequential sampling. A comparative study of several global optimization algorithms with the MEGO on the surface texture design is performed. Subsequently, surrogate-based optimization and parameter analysis are carried out, resulting in the identification of an optimal set of texture parameters. The findings reveal the superiority of the MEGO in both model prediction accuracy and refinement of minima. Moreover, compared to the base design, the friction coefficient can be reduced by up to 45.2%.

## Introduction

**Figure 1:**
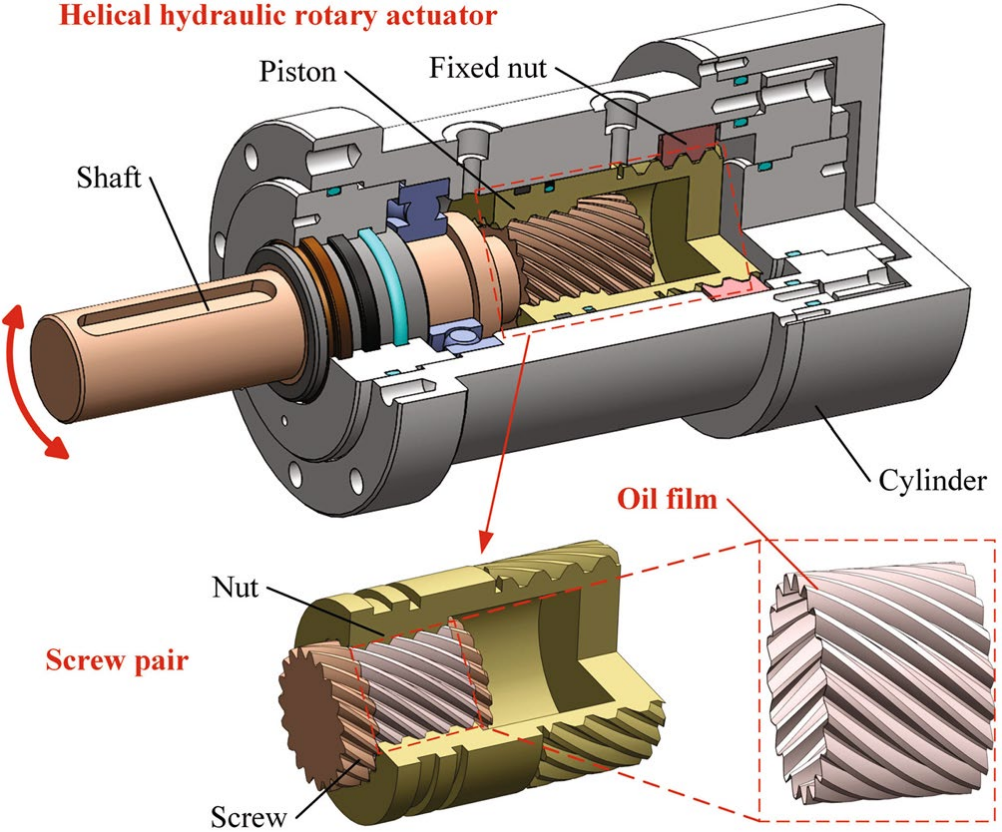
General configuration of HHRA.

The helical hydraulic rotary actuator (HHRA), a hydraulic component encapsulating two helicalpairs within a cylinder, boasts several advantages, including high efficiency, light weight, compact size, and substantial torque. Furthermore, it is capable of outputting a large angle exceeding 360°^1^. As depicted in Fig. 1, the primary components of a two-stage HHRA are a piston, a fixed nut, a shaft, and a cylinder. The piston is activated by the reciprocating motion through the gear meshing of the helicalpairs, precipitated by the differential pressure between the inlet and outlet, which in turn compels the shaft to rotate both clockwise and counterclockwise, thereby outputting a specific angle and torque.

Generally, the HHRA operates under conditions of low speed and heavy load. Under these circumstances, the lubrication regime between the helical pair predominantly falls within the mixed or boundary lubrication, leading to contact between rough surfaces. Such suboptimal lubrication conditions can exacerbate the friction and wear of the helical pair, detrimentally affecting the transmission efficiency, reliability, and service life of the HHRA. Consequently, the reduction of friction within the helical pair, aimed at mitigating sliding wear and boosting the transmission efficiency of the HHRA, is of paramount importance.

Surface texturing technology serves as a crucial tool for enhancing the lubrication conditions of friction pairs. As a simple, cost-effective, and efficient method for improving tribological performance, it has seen widespread adoption in various tribosystems. Numerous successful instances have demonstrated that surface texturing, including dimples and grooves, offers advantages such as secondary lubrication, wear particle accommodation, and supplementary hydrodynamic lubrication^2^. When implemented in helical pairs, surface texture can demonstrate anti-friction properties, potentially shifting a mixed lubrication regime to a hydrodynamic one during specific operational periods. However, the design of an optimal surface texture is complicated by two principal factors. First, the consideration of a broad array of design variables is necessary. For instance, in the case of microscale dimples, parameters like size, distribution, and contour shape can significantly affect the load-bearing capacity and friction coefficient of the contact surface. An improperly designed surface texture could undermine its tribological properties^2^. Second, the mathematical model for a helical pair is more intricate than those for conventional planar contact pairs. While many studies assume a constant oil film thickness between contact surfaces for certain lubricating interfaces, such as thrust bearings, facilitating the direct establishment of lubrication models based on the Reynolds equation or the computational fluid dynamics (CFD) method, the helical pair is subjected to mixed lubrication, necessitating the consideration of asperity contact. The helical shape of the oil film in a helical pair further necessitates transformation into an equivalent plane, introducing additional structural parameters and increasing the complexity of the mathematical model. Lastly, the oil film thickness between helical pairs, primarily dependent on the external load, is yet to be definitively determined and requires numerous iterative calculations, rendering the design procedure more time-consuming and challenging to execute.

With their independence from gradient information and ease of implementation, meta-heuristic algorithms have emerged as an efficacious approach to texture optimization. Wang et al.^3^ used a hybrid evolutionary algorithm for the optimization design of texture bottom contours, demonstrating that global-optimum bottom contours exhibited superior load capacity compared to regular ones. Similarly, Zhang et al.^4^ introduced an optimal design scheme for surface texture based on a genetic algorithm (GA), successfully enhancing the unidirectional sliding tribological performance. Their results indicated that fish and bullet shapes delivered optimal tribological performance. Chen et al.^5^ utilized a nondominated sorting genetic algorithm II (NSGA-II) to optimize the surface texture at the interface between the cylinder and valve plate in an axial piston pump, with simulation results showing that water-drop-shaped dimples displayed superior tribological behaviors and could reduce leakage by an average of 9.9%. Tang et al.^6^ employed a multi-objective hybrid evolutionary algorithm to maximize load carrying capacity while minimizing the friction coefficient in an axial piston pump by optimizing the surface texture of the slipper bearing, achieving improvements of 64.8% and 4.5% respectively after optimization. Bei et al.^7^ utilized the average Reynolds equation to solve the rough contact model and employed GA to ascertain the optimal texture shape. While these methods have been widely and successfully implemented, they necessitate extensive calculations and iterations during the optimization process. This drawback renders them suitable only for the optimization of surface texture that involves short-duration simulations and a limited number of design variables.

Surrogate modeling methods, such as support vector regressions (SVR)^8^, Kriging models^9, 10^, and radial basis functions (RBF)^11^, employ fast-computation surrogate models to fit time-consuming simulations, thereby significantly reducing the optimization process. Within the realm of engineering, there exist many successful instances where the approximate method is employed to simulate expensive numerical simulations for model prediction^12–14^, subsequently leading to the proliferation of numerous surrogate-based optimization algorithms^15–17^. Among them, the efficient global optimization (EGO) algorithm^18^ is frequently utilized due to its high efficiency and global optimization capability with limited sample points. The EGO is one of the most widely studied and applied surrogate-based optimization algorithms, with its applications primarily in aerodynamics. Jeong, Murayama, and Yamamoto^19^ introduced a novel global optimization method that combines EGO and genetic algorithm, suitable for aerodynamic design. Ghoreyshi, Badcock, and Woodgate^20^ presented a hybrid sampling strategy that combines maximizing the mean square error(MAXMSE) criterion and expected improvement (EI) function within the EGO framework, which was applied to the generation of aerodynamic tables for flight simulation. Ariyarit et al.^21^ demonstrated a hybrid surrogate-based optimization method premised on standard EGO for aerodynamic optimization, showcasing the capacity of the method to generate superior blade shapes with improved aerodynamic efficiency. Guzman Nieto, ElSayed, and Walch^14^ introduced an EGO-based algorithm to effectively predict critical dynamic aeroelastic loads, with the results indicating a total time reduction of 40.79% compared to the sole use of Kriging interpolation. Despite EGO’s commendable performance in solving these optimization problems, its deployment in texture design necessitates further exhaustive investigation. Certain constraints limit its usefulness: EGO primarily centers around local search, potentially leading to convergence to a local optimum as opposed to a global one when encountering potent nonlinear problems^22^. This focus can also result in poor model precision when sample points are limited in high-cost computation scenarios. Thus, the development and application of a suitable surrogate-based optimization algorithm for the multi-variable, strongly nonlinear model of surface texture warrants further exploration.

To reduce the friction of helical pair in HHRA by reasonable surface texture design, and improve the efficiency of the optimization design process, a modified efficient global optimization method, which takes into account the shortcomings of the standard EGO algorithm, is proposed. The first step in this method constitutes the construction of an initial Kriging model, with the OLHS strategy being employed to procure a set of uniform sample points for the establishment of the Kriging model. The second step involves the addition of new infilling samples to seek the global minimum and enhance model precision with minimal expenditure; a modified expected-improvement sampling criterion, capable of adaptively selecting the infilling samples in accordance with the optimization process, is utilized as the sequential sampling approach. Following this, the mathematical model of the textured helical pair under the mixed lubrication regime is established, based on which the proposed MEGO is utilized to construct a surrogate model for the low friction coefficient design of the surface texture. To demonstrate the superiority of MEGO in surface texturing design, a comparative study involving several global optimization algorithms and the MEGO with respect to surface texture design is executed. Lastly, surrogate-based optimization and analysis are conducted, resulting in the identification of an optimum set of texture parameters of the elliptical-shape dimples. Parameter analysis is then undertaken using the global sensitivity analysis method, correlation analysis, and controlled variable method.

## Optimization design methodology

### Optimized Latin hypercube sampling

In 1979, McKay, Beckman, and Conover^23^ originally proposed Latin hypercube sampling(LHS). Afterward, Stein^24^ proposed a mathematical approach to this method, it can be expressed as an $N \times M$ matrix *Q*:

1 $$\begin{aligned} \begin{aligned} Q = \left[ {\begin{array}{*{20}{c}} {{Q_1}}\\ {{Q_2}}\\ \vdots \\ {{Q_N}} \end{array}} \right] {\mathrm{= }}\left[ {\begin{array}{*{20}{c}} {{q_{11}}}&{}{{q_{12}}}&{} \cdots &{}{{q_{1M}}}\\ {{q_{21}}}&{}{{q_{22}}}&{} \cdots &{}{{q_{2M}}}\\ \vdots &{} \vdots &{} \ddots &{} \vdots \\ {{q_{N1}}}&{}{{q_{N2}}}&{} \cdots &{}{{q_{NM}}} \end{array}} \right] \end{aligned} \end{aligned}$$

where *N* is the number of selected points, $Q_i$ is the *i*th sample point, $q_{ij}$ is the value of the *j*th dimension of the *i*th sample point.

LHS employs multi-dimensional stratified sampling to fill the entire design space without overlap. Despite this, LHS suffers from inadequate spatial uniformity, making it challenging for initial sample points to capture the global information of the actual model. To address this, Liefvendahl and Stocki^25^ devised an optimization criterion function to enhance the distribution uniformity of samples. The objective function of this optimized Latin hypercube sampling (OLHS) criterion takes the following form:

2 $$\begin{aligned} G(Q) = \sum \limits _{i = 1}^N {\sum \limits _{j = i + 1}^N {\frac{1}{{{{\left\| {{Q_i} - {Q_j}} \right\| }^2}}}} } \end{aligned}$$

### Kriging model

The Kriging model, a renowned surrogate model based on the Gaussian process, has found widespread use across numerous engineering fields, particularly following its application to computer simulation by Sacks et al.^26^. An ordinary Kriging model can be articulated as follows:

3 $$\begin{aligned} \hat{f}(\textbf{x})=\mu +\epsilon (\textbf{x}) \end{aligned}$$

where $\mu$ denotes the mean value of the Kriging prediction function, while $\epsilon (\textbf{x})$ is the error term of a Gaussian process with zero mean and nonzero variance. The covariance of this term can be defined as:

4 $$\begin{aligned} {\text {Cov}}\left( \epsilon \left( x^{i}\right) , \epsilon \left( x^{j}\right) \right) = \varvec{R}\left( \left[ c\left( x^{i}, x^{j}\right) \right] \right) \end{aligned}$$

where $c\left( x^{i}, x^{j}\right)$ is a correlation function primarily influenced by the distance between the sample points $x^{i}$ and $x^{j}$, and $\varvec{R}()$ denotes the symmetric correlation matrix.

While various forms can be adopted for $\phi \left( x^{i}, x^{j}\right)$, including exponential, Gaussian, cubic, and others, this study employs the most commonly used Gaussian form:

5 $$\begin{aligned} R\left( x^{i}, x^{j}\right) =\exp \left[ \sum _{k=1}^{num} \theta _{k}\left| x_{k}^{i}-x_{k}^{j}\right| ^{2}\right] \end{aligned}$$

where *num* signifies the number of variables, and $\theta _{k}$ represents a correlation parameter, previously unascertained, that can be computed by resolving the maximum likelihood function.

The predictive value $\hat{f}$ for untried sample point $\textbf{x}$ within the Kriging model can be conveyed as:

6 $$\begin{aligned} \hat{f}\left( \textbf{x}\right) =\mu \left( \textbf{x}\right) +\epsilon \left( \textbf{x}\right) =\hat{\mu }+\textbf{r}^{T} \textbf{R}^{-1}(\textbf{y}-\textbf{1} \hat{\mu }) \end{aligned}$$

The Kriging variance, also known as the mean squared error (MSE), is expressed as follows:

7 $$\begin{aligned} s^2(\textbf{x})=\hat{\sigma }^2\left[ 1-\textbf{r}^{\textrm{T}} \textbf{R}^{-1} \textbf{r}+\frac{\left( 1-\textbf{1}^{\textrm{T}} \textbf{R}^{-1} \textbf{r}\right) ^2}{\textbf{1}^{\textrm{T}} \textbf{R}^{-1} \textbf{1}}\right] \end{aligned}$$

in this equation, $\textbf{y}=\left[ y_{1}, y_{2} \ldots y_{n}\right] ^{T}$, where $y_{1}, y_{2} \ldots y_{n}$ constitute the responses of the *i*th sample point. $\textbf{1}=[1, 1 \ldots 1]^{T}$, and $\textbf{r}=\left[ c_{, 1} c_{, 2} \ldots c_{*, n}\right] ^{T}$. The estimates of $\mu$ and $\sigma ^{2}$, denoted by $\hat{\mu }$ and $\hat{\sigma }^{2}$, are provided as follows:

8 $$\begin{aligned} \hat{\mu }=\frac{\textbf{1}^{\textrm{T}} \textbf{R}^{-1} \textbf{y}}{\textbf{1}^{\textrm{T}} \textbf{R}^{-1} \textbf{1}} , \quad \hat{\sigma }^{2}=\frac{(\textbf{y}-\textbf{1} \hat{\mu })^{\textrm{T}} \textbf{R}^{-1}(\textbf{y}-\textbf{1} \hat{\mu })}{n} \end{aligned}$$

### Modified expected improvement function

The EI function was initially introduced as a statistical criterion for Kriging-based optimization by Jones, Schonlau, and Welch^18^. This function leverages the Kriging model to glean statistical information from each sample point, guiding the selection of new infilling points and the overall search direction of the EGO algorithm.

The EI function can be expressed as follows:

9 $$\begin{aligned} \begin{aligned} \textrm{E}[I(\textbf{x})]=\left\{ \begin{array}{ll} \left( f_{\min }-\hat{f}(\textbf{x})\right) \xi \left( \frac{f_{\min }-\hat{f}(\textbf{x})}{s(\textbf{x})}\right) +s(\textbf{x}) \psi \left( \frac{f_{\min }-\hat{f}(\textbf{x})}{s(\textbf{x})}\right) &{}\quad s(\textbf{x})>0 \\ 0 &{} \quad s(\textbf{x})=0 \end{array}\right. \end{aligned} \end{aligned}$$

where $f_{\min }$ signifies the minimum value of the real function. $\psi (\cdot )$ represents a probability density function, while $\xi (\cdot )$ symbolizes a cumulative distribution function with a standard normal distribution. The expression $(\left( f_{\min }-\hat{f}(\textbf{x})\right)$ indicates a potential improvement, facilitating a more localized exploitation of the search space, whereas $s(\textbf{x})$ reflects the uncertainty inherent in the surrogate model, which is responsible for a more globally explorative search, quite the opposite. Consequently, the EI function can seamlessly balance global exploration and local exploitation within the optimization process.

While the EI function can locate an optimal value in certain simplistic cases, the computed standard deviation $s(\textbf{x})$ tends to be marginally lower than the actual value, a phenomenon often referred to as “underestimation”^22^. Consequently, only points in close proximity to the current optimal solution register significant EI values, leading to a more detailed exploration of the adjacent region until the local uncertainty decreases sufficiently, prompting a more global search.

One way to utilize the EGO framework for a more accurate surrogate model and more global search in the early stage is to modify the EI function. Sóbester, Leary, and Keane^27^ presented a weighted expected improvement criterion allowing for a more flexible means of balancing global and local search results. Ponweiser, Wagner, and Vincze^22^ presented the “generalized expected improvement” (GEI) criterion, founded on the EI criterion, where the parameter “g” determined whether the algorithm tends to local exploitation or global exploration. Despite the enhanced global search capabilities of the EGO algorithm offered by these methods, they still require manual parameter setting. In practical applications, selecting an optimal value for sampling poses a considerable challenge for designers.

Given the slight underestimation of the prediction error by the EGO algorithm in Formula9, the global reach of the EI function can be extended by incorporating a weighting factor preceding the term $s(\textbf{x})$. This factor should evolve with the iterations, as a global exploration at the onset of the EGO algorithm is preferred, while local exploitation is desirable in the latter half^22^.

To fulfil this requirement, the smooth, stepwise increasing behaviour of the sigmoid function is leveraged, and a variant sigmoid function^28^ is introduced and rewritten as the trend term for the weight coefficient. This modification enhances the global scope in the first half of the iteration, and allows the weight coefficient to gradually decrease with the iteration of the sample point. The formula is expressed as follows:

10 $$\begin{aligned} \begin{aligned} w_{\textrm{iter}}=(1-1 /((1+e^{-1 / \root 3 \of {n_{iter}}})\cdot (i_{\text {iter }}-n_{\text {iter }} e^{-5 i_{\text {iter }} / n_{\text {iter }}}))) \end{aligned} \end{aligned}$$

where $n_{\text {iter }}$ signifies the total number of sample updates, whereas $i_{\text {iter }}$ represents the $i_{\text {iter }}$th update.

**Figure 2:**
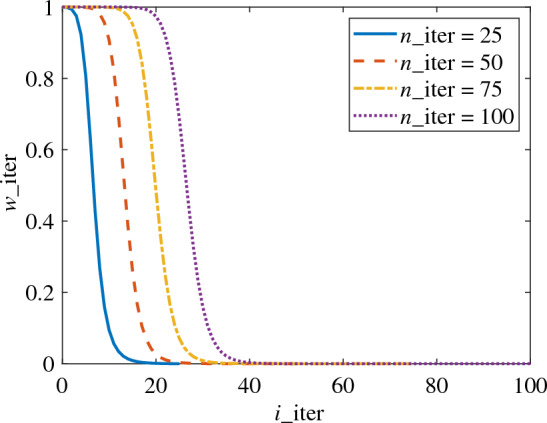
Variation of $w_{\textrm{iter}}$ with $i_{\text {iter }}$.

The correlation between $w_{\textrm{iter}}$ and $i_{\text {iter }}$ under varying values of $n_{\textrm{iter}}$ is depicted in Fig. 2. It can be observed that $w_{\textrm{iter}}$ approximates 1 in the initial third of $n_{\textrm{iter}}$ and swiftly transitions from 1 to 0 in the regions spanning from the first third to half of $n_{\textrm{iter}}$. In essence, the global weight coefficient escalates in the first half of $n_{\textrm{iter}}$, facilitating a more global search. For the latter half of $w_{\textrm{iter}}$, the weight value is nearly 0, implying that the EI algorithm is retained, thus ensuring a more localized search in the second half of the optimization process.

Additionally, $\theta _{k}$ in Formula5 emerges as a critical parameter of the Kriging model. A larger $\theta _{k}$ value augments the fluctuation of the Kriging model curve, while a smaller $\theta _{k}$ value smooths the curve. Greater values of $\theta _{k}$ suggest the need for a more global search, and the converse is also true. Therefore, $\theta _{k}$ serves as an excellent choice for a weighting regulator to adjust the weight ratio. As a vector, $\theta _{k}$ can be quantified via the introduction of the Euclidean norm and normalized using the $\arctan$ function.

Applying these principles, the weighting regulator $w_{\theta _{k}}$ can be formulated as follows:

11 $$\begin{aligned} w_{\theta_\text{k}}=\arctan \left( \left\| \theta _{k}\right\| _{2}\right) \end{aligned}$$

Therefore, the MEI function can written as:

12 $$\begin{aligned} \begin{aligned} \textrm{ME}[I(\textbf{x})]=\left\{ \begin{array}{l} (1+w_{\textrm{iter}}w_{\theta _{k}})s(\textbf{x}) \psi \left( \frac{f_{\min }-\hat{f}(\textbf{x})}{s(\textbf{x})}\right) +\left( f_{\min }-\hat{f}(\textbf{x})\right) \xi \left( \frac{f_{\min }-\hat{f}(\textbf{x})}{s(\textbf{x})}\right) \quad \quad \ \ s(\textbf{x})>0\\ \qquad \qquad \qquad \qquad \qquad \qquad 0 \qquad \qquad \qquad \qquad \qquad \qquad \qquad \quad s(\textbf{x})=0 \end{array}\right. \end{aligned} \end{aligned}$$

The MEI is correlated with $i_{\text {iter }}$ and $\theta _{k}$, changing in accordance with updates to the Kriging model. This correlation facilitates a more global search during the initial stages and a more localized search near the end of an iteration, with the weight ratio adapting in tandem with updates to the Kriging model.

## Mathematical model

### Geometry and governing equations

**Figure 3:**
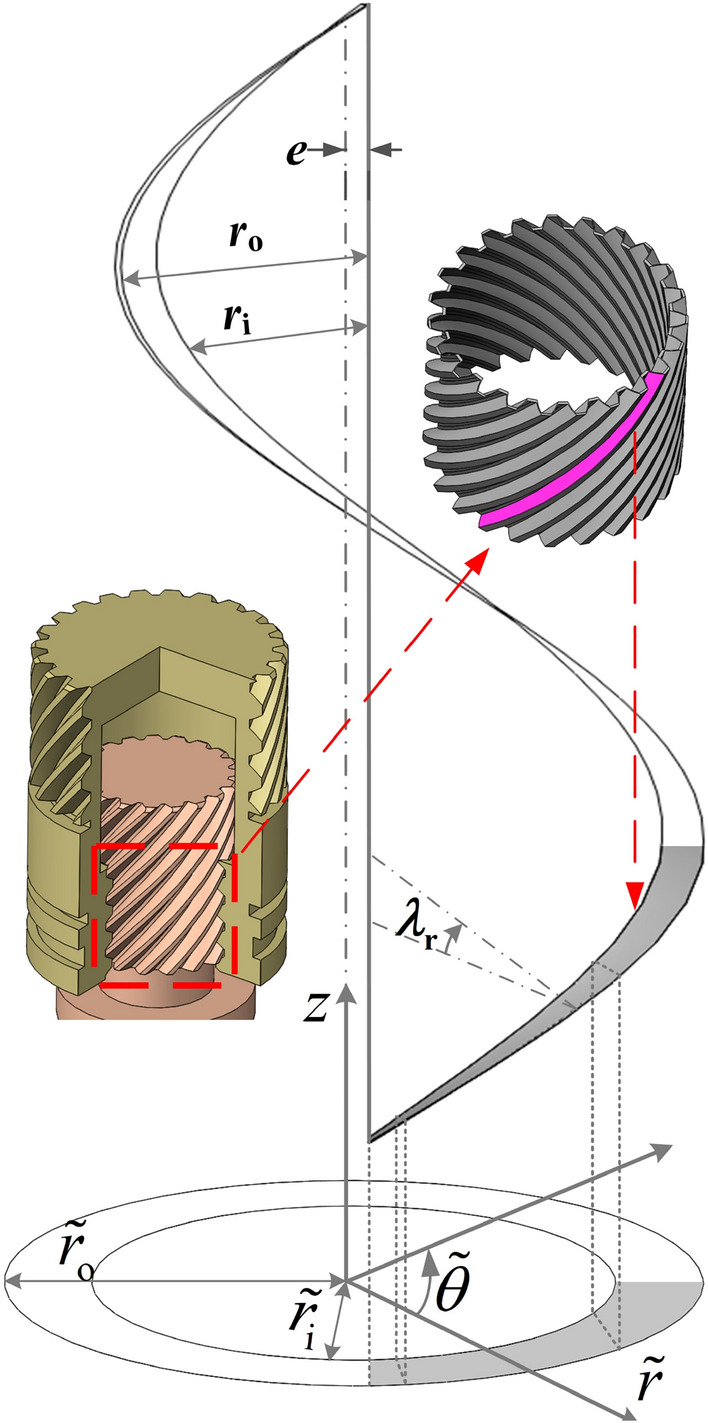
Equivalent section of the interface of helical pair.

The equivalent section of the interface of the helical pair, as depicted in Fig. 3, is derived using the method proposed by El-Sayed and Khatan^29^. The geometric relationship between the actual model and the equivalent model can be expressed as follows:

13 $$\begin{aligned} \tilde{\theta }_{\textrm{m}}=2 \pi K, \quad \tilde{r}_{\textrm{i}}=r_{\textrm{i}} K_{\textrm{i}}, \quad \tilde{r}_{\textrm{o}}=r_{\textrm{o}} K_{\textrm{o}}, \quad \tilde{r}=r+e \end{aligned}$$

where symbol $\tilde{}$ represents the variables are located on the equivalent plane, $\tilde{\theta }_{\textrm{m}}$ is the equivalent angle from the start to the end of helicoid. $r_{\textrm{i}}$ is the inside radius of nut, $r_{\textrm{o}}$ is the outside radius of screw, and $K=\cos \alpha \left[ \sec \lambda _{0}-\left( r_{\textrm{i}}^{2}/ r_{\textrm{o}}^{2}+\tan ^{2} \lambda _{\textrm{o}}\right) ^{1 / 2}\right] /\left( 1-r_{\textrm{i}} / r_{\textrm{o}}\right)$, $e=r_{\textrm{o}}\left[ \left( r_{\textrm{o}}-r_{\textrm{j}}\right) /\left( r_{\textrm{o}}-r_{1} \cos \lambda _{\textrm{o}} / \cos \lambda _{\textrm{i}}\right) -1\right] / \cos \alpha$, $K_{\textrm{i}}=1 / K \cos \lambda _{\textrm{i}}$, $K_{\textrm{o}}=1 / K \cos \lambda _{\textrm{o}}$.

**Figure 4:**
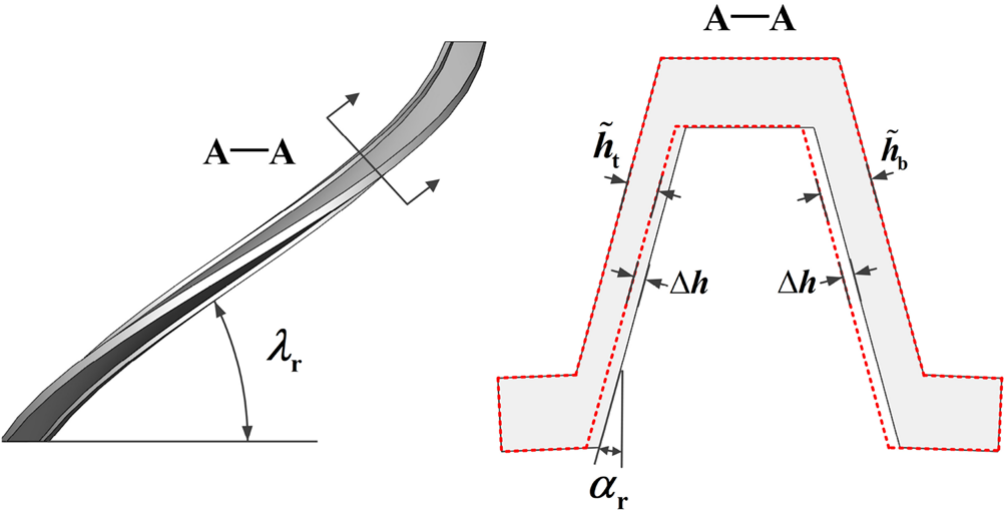
Cross-section of the screw tooth.

Figure 4 shows the cross-section of the screw tooth. Based on the equivalent model, the axial film thicknesses at any radius *r* can be written as follows:

14 $$\begin{aligned} \left\{ \begin{array}{l} \tilde{h}_{\textrm{t}}=\left( h_{\textrm{a} 0}-\Delta h\right) \cos \lambda _{r} \cos \alpha _{r} \\ \tilde{h}_{\textrm{b}}=\left( h_{\textrm{a} 0}+\Delta h\right) \cos \lambda _{r} \cos \alpha _{r} \end{array}\right. \end{aligned}$$

where $h_{\textrm{a}0}$ is the assumed axial clearance, $\Delta h$ is the clearance of screw and nut along the axial direction, the subscripts $\mathrm t / \mathrm b$ represents the oil film on the top/bottom side. $\lambda _{r}=\arctan (P/(2\pi r))$, where $\lambda _{\textrm{r}}$ denotes the helix angle at any radius *r*, *P* denotes the screw lead. $\alpha _{\textrm{r}}=\arctan \left( \tan \alpha \cos \lambda _{\textrm{r}}\right)$, in which $\alpha$ is pressure angle.

**Figure 5:**
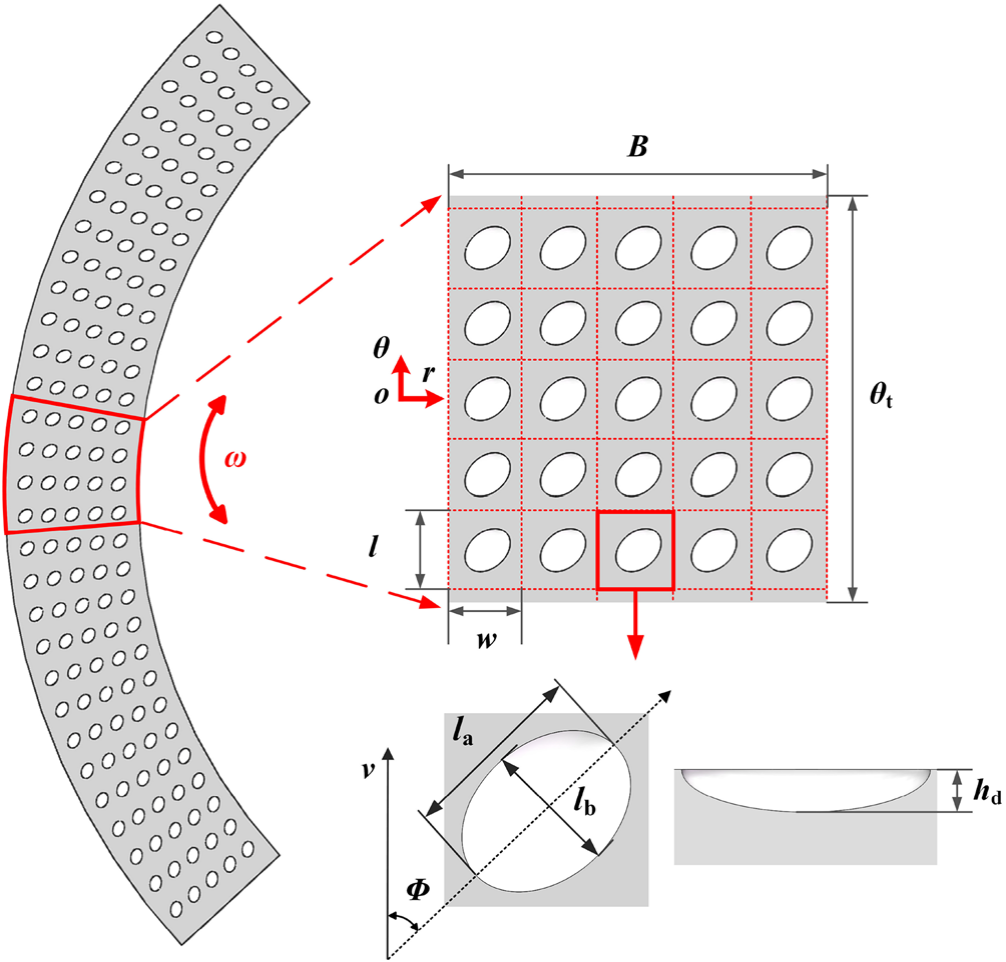
Equivalent section of the interface of helical pair with elliptical-shape dimples.

Building on the findings of Yu, Wang, and Zhou^30^, elliptical-shaped dimples deliver the optimal load-carrying capacity compared to other regular-shaped dimples such as circles, triangles, and rectangles. As a result, this study employs elliptical-shaped dimples as the textured structure of the helical pair. The representative section of the interface of the helical pair with elliptical-shaped dimples is illustrated in Fig. 5, where the dimples are uniformly distributed in both radial and circumferential directions.

To facilitate calculation, it is assumed that the texture is positioned within a rectangular unit of dimensions $l \times w$. The parameters defining the dimples are as follows: $Z_{\textrm{c}}$ represents the number of circumferential dimples, $Z_{\textrm{r}}$ the number of radial dimples, and $\phi$ the orientation of the major axis of ellipse. *B* denotes the length of the texture area in the radial direction, while $\theta _{\textrm{t}}$ signifies the length of the texture area in the circumferential direction. The dimple depth is $h_{\textrm{d}}$, with $l_{\textrm{a}}$ and $l_{\textrm{b}}$ specifying the major and minor axes of the ellipse, respectively. The spacing between dimples in the radial (*l*) and circumferential (*w*) directions, the area ratio $S_{\textrm{p}}$, the axial ratio of the ellipse $r_\text{t}$, and the texture region can be respectively expressed as follows:

15 $$\begin{aligned} \begin{aligned} l&=\frac{\theta _{\textrm{t}}}{Z_{\textrm{c}}},\quad w=\frac{B}{Z_{\textrm{r}}},\quad S_{\textrm{p}}=\frac{\pi r_{\textrm{d}}}{l w},\quad r_\text{t}=\frac{l_{\text{a}}}{l_{\text{b}}},\\ h&= {h_{\text{d}}}\sqrt{\begin{array}{l} 1 - {\left[ {\frac{{(x - l/2)\cos \phi - (y - w/2)\sin \phi }}{{{l_{\text{a}}}}}} \right] ^2} - {\left[ {\frac{{(x - l/2)\sin \phi + (y - w/2)\cos \phi }}{{{l_{\text{b}}}}}} \right] ^2} \end{array}} \end{aligned} \end{aligned}$$

Evidence has shown that provided the Reynolds number is adequately low and the aspect ratio of the dimples is sufficiently large, the Reynolds equation proves effective^31^, a condition applicable to our model. The assumption is that the oil film represents an incompressible Newtonian and laminar flow. Consequently, the equivalent Reynolds equation^32^ can be expressed as:

16 $$\begin{aligned}\frac{\partial }{\partial \tilde{r}}\left( \tilde{r} \tilde{h}_{\text{t} / \text{b}}^{3} \frac{\partial \tilde{p}}{\partial \tilde{r}}\right) +\frac{1}{\tilde{r}} \frac{\partial }{\partial \tilde{\theta }}\left( \tilde{h}_{\text{t} / \text{b}}^{3} \frac{\partial \tilde{p}}{\partial \tilde{\theta }}\right) &=12 \rho \omega ^{2} \cos \alpha \frac{\partial }{\partial \tilde{r}}\left( \frac{\tilde{r} r^{2} \tilde{h}_{\text{T} / \text{B}}^{3} \Delta _{\text{r}}}{r_{\text{c}}}\right) +6 \eta \frac{r \omega }{\cos \lambda _{\text{r}}} \frac{\partial \tilde{h}_{\text{t} / b}}{\partial \tilde{\theta }}\\&\quad \mp 12 \eta \tilde{r}\left( \frac{P}{2 \pi } \omega -\dot{z}_{\text{rut}}\right) \cos \lambda _{\text{r}} \cos \alpha _{\text{r}} \end{aligned}$$

where $\Delta _{\text{r}}=\left( 1.5-5 \sin ^{2} \lambda _{r}+5 \sin ^{4} \lambda _{r}\right) /\left( 60 \cos ^{2} \lambda _{r}\right)$, in this context, $\tilde{r}$ and $\tilde{\theta }$ are the coordinate directions of the polar coordinates $\tilde{o}-\tilde{r} \tilde{\theta }$, situated on the equivalent plane of the helicoid. The hydrodynamic pressure is denoted by *p*, with $\rho$ representing the oil density and $\eta$ its viscosity. The rotational speed is given by $\omega$, and $z_{\text {nut }}$ signifies the axial displacement disturbances. The subscript t/b refers to the top side/bottom side.

Generally, the HHRA operates under high load and low-speed conditions, resulting in a relatively thin film between the helical pair, necessitating the consideration of asperity contact force. This paper employs the Greenwood and Tripp model^33^, which is widely used in asperity contact studies. By assuming a Gaussian distribution for the height of the micro-convex bodies on the friction surface, the asperity contact pressure can be defined as:

17 $$\begin{aligned} P_{\text {asp }}\left( H_{\sigma }\right) =\frac{16}{15} \sqrt{2} \pi (A \beta \sigma )^{2} E^{\prime } \sqrt{\frac{\sigma }{\beta }} F_{\text{n}}\left( H_{\sigma }\right) \end{aligned}$$

where $E^{\prime }=E_{1} E_{2} /\left[ E_{2}\left( 1-v_{1}^{2}\right) +E_{1}\left( 1-v_{2}^{2}\right) \right]$, where $E_{1}$ and $E_{2}$ denote the elastic modulus of the two bodies in contact, and $E^{\prime }$ is the composite elastic modulus. The Poisson’s ratios of the contacting bodies are represented by $v_{1}$ and $v_{2}$. The asperity curvature radius is indicated by $\beta$, while *A* signifies the asperity density. The composite surface roughness is designated by $\sigma$, and $H_{\sigma }$ is defined as the ratio of the mean film thickness to $\sigma$.

The statistical function $F_{n}(u)$ can be written as:

18 $$\begin{aligned} F_{2.5}\left( H_{\sigma }\right) =\frac{1}{\sqrt{2 \pi }} \int _{H_{\sigma }}^{\infty }\left( s-H_{\sigma }\right) ^{2.5} e^{\frac{s^{2}}{2}} d s \end{aligned}$$

where *s* is the correlation parameter, set at 6.804^33^.

Implementing the average flow model^34^, the equivalent Reynolds equation that takes into account roughness effects can be articulated as:

19 $$\begin{aligned}\frac{\partial }{\partial \tilde{r}}\left( \tilde{r} \phi _{\text{y}} \tilde{h}_{\text{t} / \text{b}}^{3} \frac{\partial \tilde{p}}{\partial \tilde{r}}\right) +\frac{1}{\tilde{r}} \frac{\partial }{\partial \tilde{\theta }}\left( \phi _{\text{x}} \tilde{h}_{\text{t} / \text{b}}^{3} \frac{\partial \tilde{p}}{\partial \tilde{\theta }}\right) & =12 \rho \omega ^{2} \cos \alpha \frac{\partial }{\partial \tilde{r}}\left( \frac{\tilde{r} r^{2} \tilde{h}_{\text{t} / \text{b}}^{3} \Delta _{r}}{r_{\text{c}}}\right) +6 \eta \phi _{\text{c}} \frac{r \omega }{\cos \lambda _{\text{r}}} \frac{\partial \tilde{h}_{\text{T} / \text{B}}}{\partial \hat{\theta }} \\&\quad +6 \eta \sigma \frac{r \omega }{\cos \lambda _{\text{r}}} \frac{\partial \phi _{\text{s}}}{\partial \tilde{\theta }} \mp 12 \eta \tilde{r}\left( \frac{P}{2 \pi } \omega -\dot{z}_{\text {nut }}\right) \cos \lambda _{r} \cos \alpha _{r} \end{aligned}$$

where $\phi _{\text{x}}$ and $\phi _{\textrm{y}}$ represent the pressure flow factors along the x and y directions, as defined by Patir and Cheng^34, 35^. The contact factor is denoted by $\phi _{\textrm{c}}$, while $\phi _{\textrm{s}}$ signifies the shear flow factor, as outlined by Wu and Zheng^36^. The lead of the screw/nut is represented by *P*.

### Boundary conditions

#### Velocity boundary

Utilizing velocity decomposition, the velocities along the top and bottom sides of the screw tooth can be determined as follows:

20 $$\begin{aligned} \begin{aligned} v_{\tilde{\theta }_{1} / \tilde{\theta }_{2}}&=-r \omega \tan \lambda \sin \lambda _{r} \\ v_{\tilde{\theta }_{2} / \tilde{\theta }_{1}}&=r \omega \cos \lambda _{r} \\ v_{\tilde{z}_{1} / \tilde{z}_{2}}&=v_{n 1}=-\dot{z}_{\text {mut }} \cos \lambda _{r} \cos \alpha _{r} \\ v_{\tilde{z}_{2} / z_{1}}&=v_{n 2}=-\frac{P}{2 \pi } \omega \cos \lambda _{r} \cos \alpha \end{aligned} \end{aligned}$$

where $v_{\tilde{\theta }{1}/ \tilde{\theta }{2}}$ and $v_{\tilde{z}{1}/ \tilde{z}{2}}$ denote the speeds in the $\tilde{\theta }$ and $\tilde{z}$ directions, respectively.

#### Pressure boundary

The external pressure of the helical pair equals 0.1 MPa, and the pressure between the contact surface of the helical pair corresponds to the oil film load capacity. Besides, the minimum value of cavitation pressure is specified as 0.03 MPa^5^.

#### Cavitation boundary conditions

Owing to its high accuracy and ease of implementation, the Reynolds cavitation boundary condition has been effectively utilized in numerous studies investigating surface texture^4, 5, 30, 37^. Therefore, the Reynolds cavitation boundary condition is chosen in this paper.

### Calculation of friction coefficient

The expression of friction coefficient can be written as:

21 $$\begin{aligned} \mu _{\textrm{cof}}=\frac{f}{D} \end{aligned}$$

where, the total load capacity *D* is an amalgamation of the load-carrying capacity induced by hydrodynamic support and that induced by asperity contact. This can be calculated as follows:

22 $$\begin{aligned} {D_1} = \int _0^{{{\tilde{\theta }}_{\textrm{m}}}} {\int _{{{\tilde{r}}_{\textrm{i}}}}^{{{\tilde{r}}_{\textrm{o}}}} {\left( {{{\tilde{p}}_{\textrm{t}}} - {{\tilde{p}}_{\textrm{b}}}} \right) } } \cos {\lambda _{\textrm{r}}}\cos \alpha \tilde{r}d\tilde{\theta }d\tilde{r} + \int _0^{{{\tilde{\theta }}_{\textrm{m}}}} {\int _{{{\tilde{r}}_{\textrm{i}}}}^{{{\tilde{r}}_{\textrm{o}}}} {\left( {{{\tilde{p}}_{{\textrm{asp}}}}} \right) } } \cos {\lambda _{\textrm{r}}}\cos \alpha \tilde{r}d\tilde{\theta }d\tilde{r} \end{aligned}$$

The total friction force *f* comprises both the hydrodynamic friction force and the asperity contact friction force. It can be represented as:

23 $$\begin{aligned} \begin{aligned} =F = - \int _0^{{{\tilde{\theta }}_{\textrm{m}}}} {\int _{{{\tilde{r}}_{\textrm{i}}}}^{{{\tilde{r}}_{\textrm{o}}}} {\left[ {\frac{{\eta \omega \tilde{r}}}{{\tilde{h}}}\left( {{\phi _{\textrm{f}}} + {\phi _{{\textrm{fs}}}}} \right) + {\mu _{\textrm{asp}}}{P_{{\textrm{asp }}}}} \right] } } \tilde{r}d\tilde{\theta }d\tilde{r} \end{aligned} \end{aligned}$$

where *v* denotes the linear velocity, while $\phi _{\textrm{f}}$ and $\phi _{\textrm{fs}}$ indicate the flow factors induced by friction, as defined in the studies by Patir and Cheng^34, 35^. The asperity contact friction coefficient is represented by $\mu _{\text {asp }}$.

**Figure 6:**
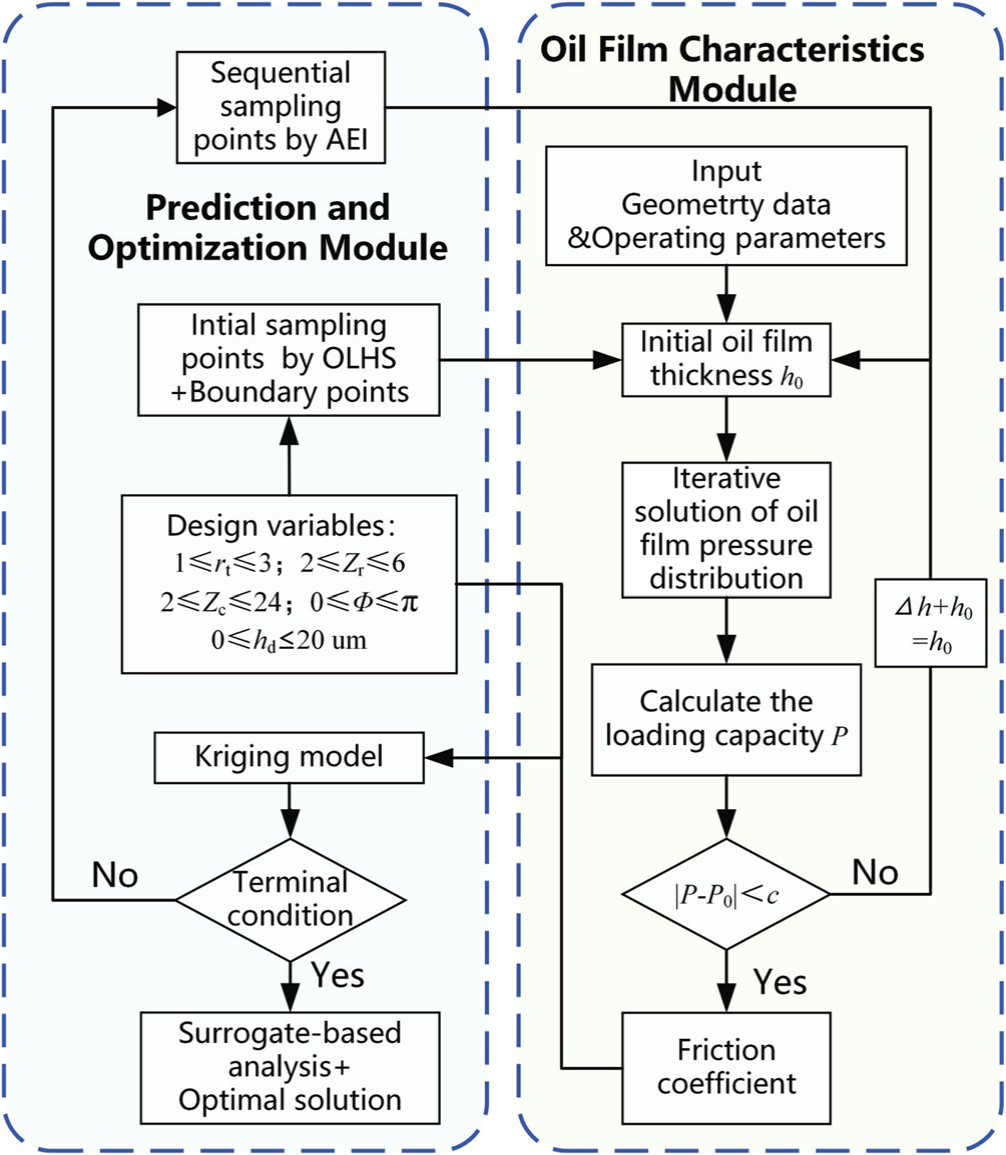
Flow chart of optimization design for the surface texture of helical pair.

For the HHRA studied in this paper, the oil film thickness between the helical pair remains undetermined. Its value is primarily influenced by the external load driven by the HHRA, necessitating computation from the external load. The comprehensive calculation procedure is depicted in the “oil film characteristics module” in Fig. 6.

The parameters used in the numerical analysis are shown in Tables 1 and 2.

**Table 1:** Geometric and operating parameters.

Geometric and operating parameters	Value	Unit
Inside radius of nut, $r_{\textrm{i}}$	33	mm
Outside radius of screw, $r_{\textrm{o}}$	40	mm
Number of teeth, $Z_{\textrm{t}}$	19	
Pressure angle, $\alpha$	15	$^{\circ }$
Helix angle, $\lambda _{\textrm{r}}$	45	$^{\circ }$
Screw lead, *P*	114.7	mm
Designed axial clearance, $h_{\textrm{a0}}$	0.15	mm
Length of screw engagement	30	mm
Oil density, $\rho$	876	kg/m$^{3}$
Deaeration pressure of dissolved air, $p_{\textrm{c}}$	0.03	MPa
Oil kinematic viscosity, $\eta$	0.04025	Pa s
Load force, *F*	65462	N
Rotational speed of the lead screw, $\omega$	120	rpm

**Table 2:** Textured surface parameters.

Textured surface parameters	Value	Unit
Composite surface roughness, $\sigma$	1.6	um
Surface pattern parameter	1	
Friction coefficient of asperity contact, $\mu _{\textrm{asp}}$	0.08	
Composite elastic modulus/, $E^{\prime }$	211	GPa
Dimple area density, $S_{\textrm{p}}$	0.14	

The equivalent Reynolds equation that incorporates roughness effects is discretized utilizing the finite difference method (FDM). To calculate the film pressure distribution, the successive over relaxation (SOR) Gauss-Seidel iterative method, with an over-relaxation factor at each discretized node, is employed. The over-relaxation factor is set between 1.5 and 1.8^37^, with a chosen value of 1.6 in this study to expedite convergence. MATLAB R2022b software facilitates the construction and resolution of the equivalent Reynolds equation, operating on a computer CPU that is i7-9750h with 16 G RAM.

Grid density significantly influences the accuracy of results. While fine-meshed grids can enhance computational accuracy, they also demand more computing time. Table 3 presents the computation time of this mathematical model at varying grid densities. Relative to the finest grid, a grid density of 120 $\times$ 720 is selected for texture optimization, as it offers the shortest computing time when the error is within 1%. However, as the computation with the 120 $\times$ 720 grid remains time-intensive, it is employed solely in the final optimization(5.2) and sensitivity analysis(5.3). For the comparison of algorithms (5.1), which necessitates the generation of a considerable number of sample points, the 40 $\times$ 240 grid is chosen. This selection is due to a greater emphasis on the alignment of the predicted value with the actual one, and the ability to locate the minimum value, rather than obtaining an accurate friction coefficient and optimal texture parameters.

**Table 3:** Computation time for several different grid densities.

Number of grids	$\mu _{\textrm{cof}}$	Elapsed time/s
20 $\times$ 120	0.0544	11
40 $\times$ 240	0.0634	48
80 $\times$ 480	0.0657	526
120 $\times$ 720	0.0678	1957
160 $\times$ 960	0.0682	4758
200 $\times$ 1200	0.0682	7817

## Optimization process

### Procedures of modified efficient global optimization

A flowchart detailing the optimization design process for the surface texture of the helical pair is provided in Fig. 6. A comprehensive explanation follows.

Step 1: Establish the optimization objectives, the ranges for design variables, and the objective and constraint functions.

Numerous studies indicate that within a certain range, the friction coefficient progressively decreases with the increasing textured area density. However, exceeding this range could elevate contact stress, limiting the reduction of the friction coefficient and intensifying wear. Xu et al.^38^ found that elliptical-shaped dimples with an area density of 10.6–14.1% yielded the best performance. To minimize the friction coefficient, a 14% area density was selected for this study. The remaining undefined geometry and distribution parameters of the elliptical-shaped dimples serve as the design parameters. Notably, most surface texture optimization research employs dimple depth and diameter as design variables for circular dimples, given their significant impact on the lubrication characteristics of surface texture. For a single texture unit, the area density directly correlates with the square of the dimple diameter. Thus, the dimple depth and diameter as design variables sufficiently encompass all necessary design information. Yet, the interaction among the dimples can also influence hydrodynamic lubrication for multiple texture units. Consequently, the axial number and the circumferential number of the dimples are chosen as part of the design variables. When the area density remains constant, these numbers not only determine the dimple diameter but also reflect the spacing between the dimples in the radial and circumferential direction.

The remaining undefined geometry and distribution parameters of the elliptical-shaped dimples function as the design parameters, with their range chosen to be technologically feasible and prevent dimple overlap.

The optimization issue concerning the surface texture of the helical pair, as considered in this study, can be expressed mathematically as follows:

24 $$\begin{aligned} {\left\{ \begin{array}{ll}\text { Find: } &{} \textbf{x}=\left\{ r_t, Z_{\textrm{r}}, Z_{\textrm{c}}, \phi , h_{\textrm{d}}\right\} ^{\textrm{T}} \\ \text { Min: } &{} y(\textbf{x})=\mu _{\textrm{cof}} \\ \text { s.t. } &{} 1 \le r_t \le 3,\,2 \le Z_{\textrm{r}} \le 6,\, 2 \le Z_{\textrm{c}} \le 24, \\ {} &{} 0 \le \phi \le \pi \, \textrm{rad}, \, 0 \le h_{\textrm{d}} \le 20 \, \textrm{um}\end{array}\right. } \end{aligned}$$

Step 2: Conduct a design space exploration and sample initial points using the design of experiment (DOE) method. In this study, the OLHS method mentioned above is used to obtain maximum information by uniformly adding sample points from the design space.

The initial number of sample points is determined by the number of variables. According to Nuchitprasittichai and Cremaschi^39^, the initial number of sample points is typically ten times the number of variables. Considering that this study involves five design variables, an initial sample count of 50 is chosen. However, due to the constraints of the OLHS method, sample points are not selected at the boundary, resulting in an incomplete coverage of the design area. The boundary points are defined by upper and lower bounds, and with five variables included in this study, the total number of boundary points is $2^5$. As a result, an aggregate of 82 (50 + $2^5$) initial sample points is generated.

Step 3: Conduct the numerical analysis as outlined in “Mathematical model”, using the initial sample points determined in Step 2.

Step 4: Use the results from Step 3 to establish the initial Kriging model, representing the mapping relationships between the objective functions and the design variables.

Step 5: Execute the sequential sampling strategy, employing the MEI function to boost the accuracy of the Kriging model and locate the minimal values of the actual model through the addition of new sample points. The sequential sampling process concludes when the count of iteration steps for sequential sampling attains the predetermined value.

To evaluate model accuracy, 50 sample points are used to calculate the root mean square error(RMSE) value of the Kriging model. The stopping criterion during the sampling process is the total number of sample points reaching 200 times. Therefore, 118 iterations are required, given that the initial number of sample points is 82.

Step 6: The final Kriging model, constructed using both initial and sequential sampling data, serves to predict the targets, effectively replacing the need for laborious numerical analysis, and enables the procurement of optimal design results.

### Evaluation index of surrogate-based optimization algorithm

Predictive accuracy and optimization potential are critical factors for surrogate-based optimization algorithms. To appraise the accuracy of the surrogate model in quantitative terms, the introduction of three evaluation indices is proposed as follows:

25 $$\begin{aligned} {\textrm{MRE}}=\frac{\sum _{i=1}^{n} \frac{\left| f_{\text {actual }}^{i}-f_{\text {predict }}^{i}\right| }{f_{\text {actual }}^{i}}}{n} \times 100 \%, \\ {\textrm{RMSE}} =\sqrt{\frac{\sum _{i=1}^{n}\left( f_{\text {actual }}^{i}-f_{\text {predict }}^{i}\right) ^{2}}{n}}, \\{\text {MaxRE}}_{i}=\left| \frac{f_{\text {actual }}^{i \max }-f_{\text {predict }}^{i \max }}{f_{\text {actual }}^{i}}\right| \times 100 \%, \end{aligned}$$

where $f_{\text {predict }}$ denotes the predicted value obtained from the surrogate model, while $f_{\text {actual }}$ refers to the actual value derived from numerical analysis.

The optimization potential is evaluated as follows:

26 $${\text {Error}}=\left| \frac{\mathrm{opt.}_{\text {actual }}-\mathrm{opt.}{\text {predict }}}{\mathrm{opt.}{\text {actual }}}\right| \times 100 \%,$$

where $\mathrm{opt.}{\text {predict }}$ refers to the globally optimized solution obtained from the surrogate-based optimization algorithm, while $\mathrm{opt.}_{\text {actual }}$ denotes the actual global optimized solution.

## Results and discussion

### Algorithm comparison

To demonstrate the superiority of the MEGO method in surface texture design, it is compared with commonly used surrogate-based optimization algorithms: EGO^18^, Kriging+GA^16^, RBF+GA^15^, BP+GA^40^, and the typical meta-heuristic algorithm GA.

**Figure 7:**
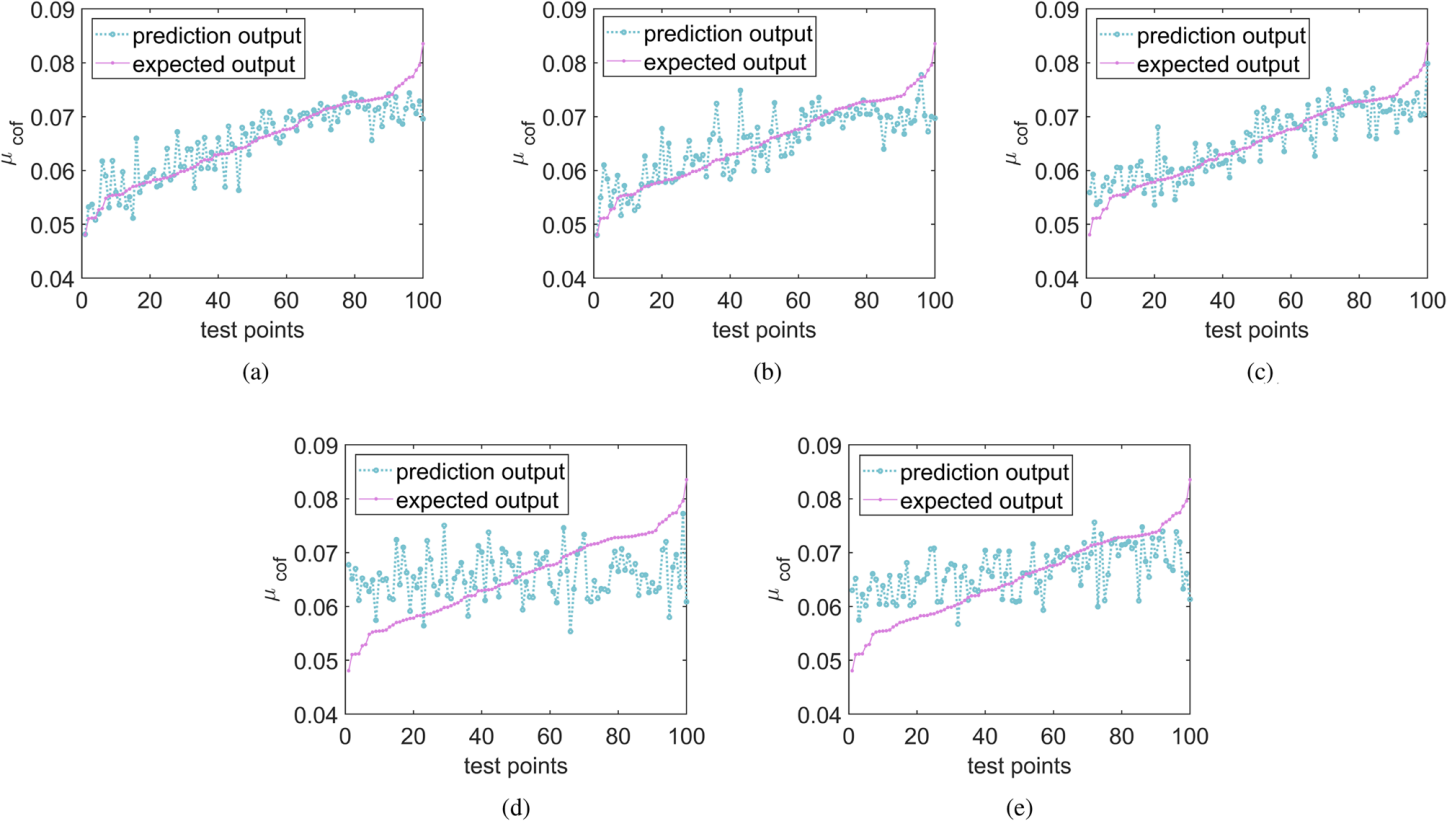
Prediction of test points (**a**) MEGO (**b**) EGO (**c**) Kriging+GA (**d**) RBF+GA (**e**) BP+GA.

**Figure 8:**
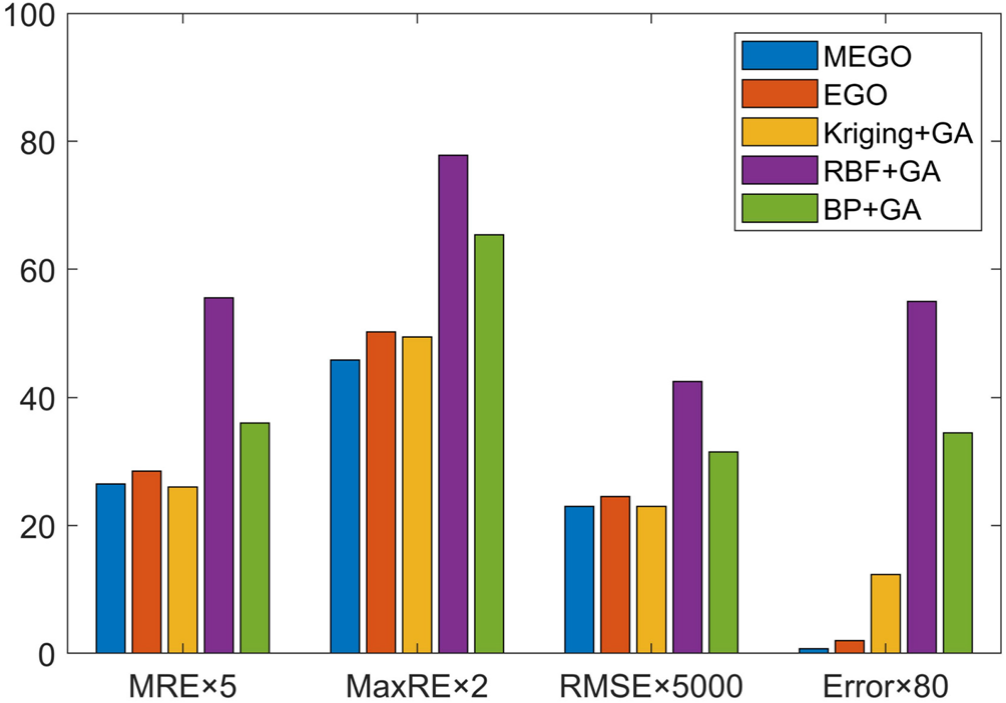
Comparison of the five different global optimization algorithm.

It is worth noting that the algorithm comparison, distinct from the optimization steps outlined in 4.1, prescribes a fewer number of initial sample points and a maximum limit for sample points. This arrangement is designed to evaluate their ability to seek optimization and fitting accuracy under the restriction of limited sample points. Hence, each global optimization algorithm is limited to 60 points, equivalent to 60 simulation times. Initially, 20 points are generated using the OLHS method, and the remaining 40 infilling points are selected by the MEI function for each iteration. To test the accuracy of the established surrogate model, 100 random test samples are generated. Given the randomness of the sampling distribution induced by DOE, each global optimization algorithm is computed 20 times for every case, and the results are averaged. The standard deviation (std) is employed here to gauge the stability of the optimization results. For ensuring the accuracy of the prediction model, MRE and MaxRE should be as minimal as possible, and a lower RMSE evaluation index signifies a more accurate prediction model. Figure 7 displays the test points predicted by each algorithm, and the comparative results are depicted in Table 4 and Fig. 8. Notably, GA, while capable of global optimization, lacks model prediction ability, hence it is excluded from discussions about the accuracy of the prediction model. The minimum $\mu _{\textrm{cof}}$ is 0.0433, as calculated by the Monte Carlo sampling method.

**Table 4:** Results of the different global optimization algorithm for texture parameters.

Global optimization algorithm	Initial Sample strategy	Sequential sampling criterion	MRE	MaxRE (%)	RMSE	$\mathrm{Opt.}_{\text {predict }}$	Error(%)	$\textrm{Std}$
MEGO	OLHS	MEI	5.3	22.9	0.0046	0.0439	1.4	0.0006
EGO	OLHS	EI	5.7	25.1	0.0049	0.0446	3.0	0.001
Kriging+GA	OLHS	–	5.2	24.7	0.0046	0.0501	15.7	0.0057
RBF+GA	OLHS	–	11.1	38.9	0.0085	0.0732	69.1	0.0145
BP+GA	OLHS	–	7.2	32.7	0.0063	0.0621	43.4	0.0083
GA	–	–	–	–	–	0.0538	24.2	0.0065

It is clearly observed that the Kriging-based optimization algorithms (MEGO, EGO, Kriging + GA) showcase lower MRE, MaxRE, and RMSE values compared to the others (RBF + GA, BP + GA). This highlights the superior ability of the Kriging-based surrogate model to fit the nonlinear curve of the actual model in this case. Moreover, the MRE, MaxRE, and RMSE values for EGO are marginally higher than those for MEGO, signaling the superior prediction ability of MEGO over the standard EGO.

Regarding the optimal value, the global optimization ability is sorted according to the magnitude of $({\mu _{\textrm{cof}}})_{\min }$ as follows: MEGO > EGO > Kriging + GA > GA > BP + GA > RBF + GA. It is evident that the global optimization ability of MEGO surpasses that of the other models, being only 1.4% away from the optimal result. In contrast, EGO, Kriging + GA, RBF + GA, BP + GA, and GA are 3.0%, 15.7%, 69.1%, 43.4%, 24.2% away from the optimum result, respectively. This implies that MEGO can yield the optimal global solution with the fewest iterations. Furthermore, MEGO exhibits the smallest standard deviation among all global optimizations, indicating that MEGO consistently delivers the most stable optimization results.

### Optimum texture parameters

**Figure 9:**
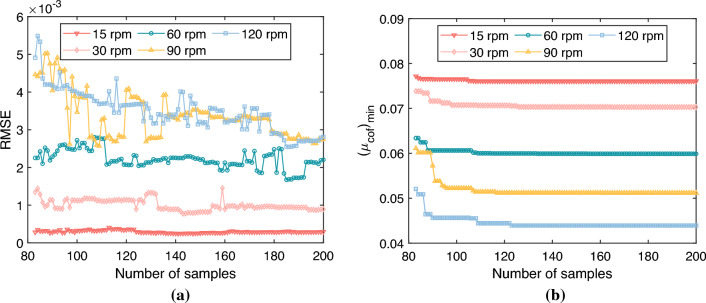
Convergence histories for various rotational speed (**a**) RMSE (**b**) Minimum friction coefficient.

Before embarking on the parameter analysis, it is crucial to confirm that the accuracy demands are fulfilled by the constituted Kriging model. At the same time, it must be assured that a local minimum does not stand as the final optimization outcome in the course of texture design optimization. To support this, the RMSE values from each step of the sampling process, obtained via the MEI method, together with the minimum friction coefficient values, are recorded in this paper. A complete visual representation is available in Fig. 9a,b.

For the friction coefficient studied in this paper, its value ranges from 0.04 to 0.09. When the RMSE value is less than 0.003, the established surrogate model can be considered to meet the engineering accuracy requirements. As discernible from Fig. 9a, the RMSE values of the Kriging model at a rotational speed less than 60 rpm remain below 0.003(the RMSE value is still less than 0.003) at all sample points, exhibiting no significant fluctuations with the increase in iteration steps (the RMSE value is still less than 0.003). This suggests that a highly accurate Kriging model can already be established from the initial sample points, with no overfitting observed upon the addition of more sample points. Moreover, the RMSE values of the Kriging models at rotational speeds of 90rpm and 120rpm initially exhibit considerable fluctuations and are greater than 0.003. However, as the number of iteration steps increases, the RMSE values of both models gradually converge to less than 0.003 after about 175 sample points and remain so at the 200th step. Hence, it can be concluded that when the number of sample points is set to 200, the Kriging model at this juncture meets the accuracy requirements. As depicted in Fig. 9b, no changes are observed in the minimum friction coefficient values as the number of iteration steps increases when the number of sample points exceeds 130. From the previous analysis of the MEI, it is recognized that the MEI is capable of balancing local development and global exploration. No further reductions in the friction coefficient are observed when the number of sample points increases from 130 to 200. Consequently, it can be inferred that the proposed algorithm has potentially located the global minimum of the friction coefficient.

**Figure 10:**
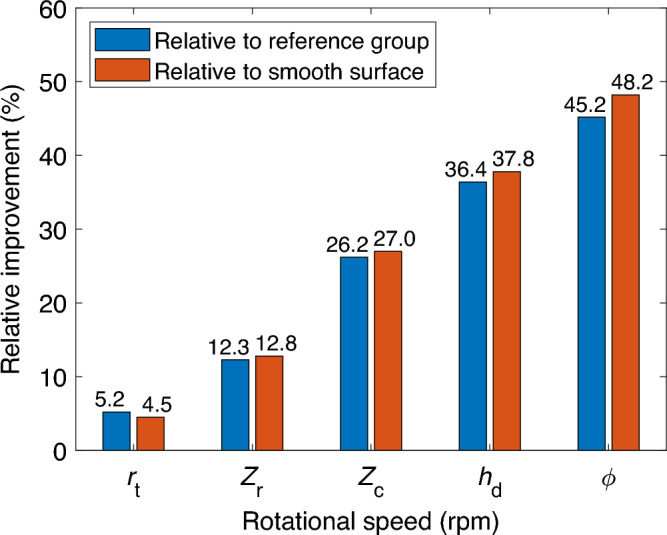
Percentage increase in friction coefficient.

**Figure 11:**
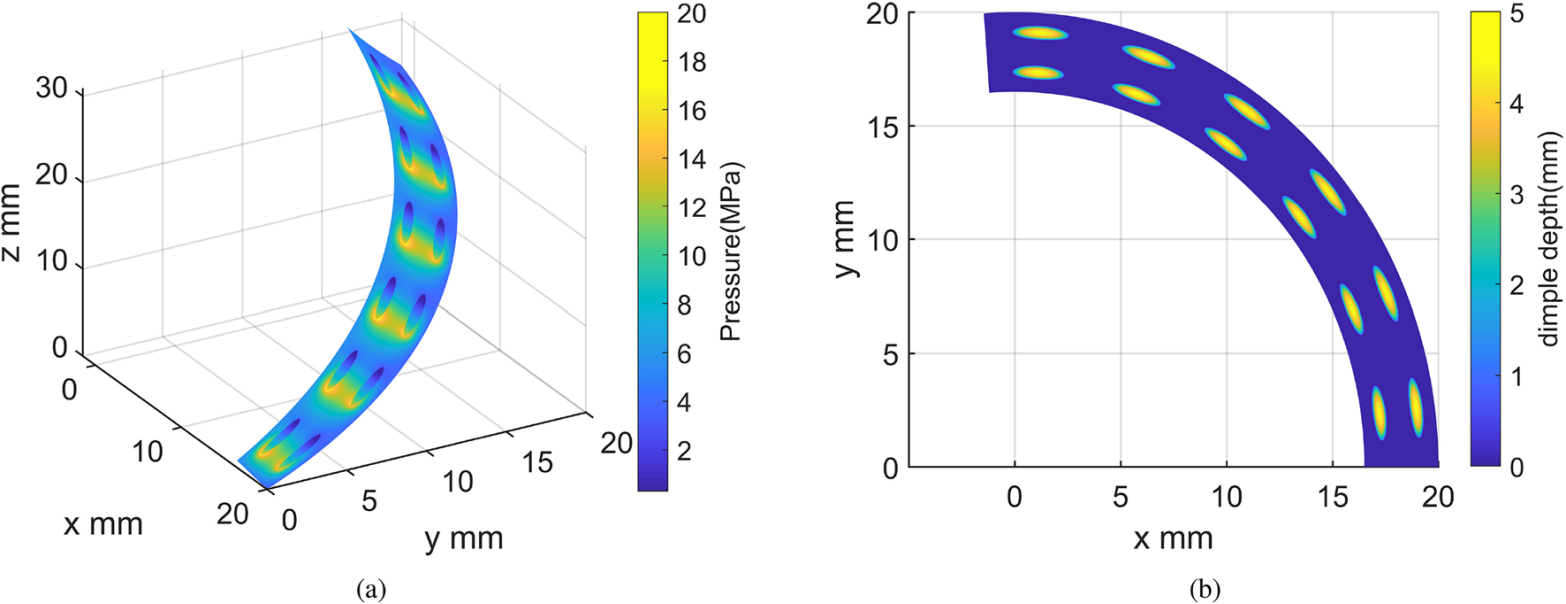
Optimum design of surface texture. (**a**) Pressure distributions. (**b**) Top view of texture profile.

**Figure 12:**
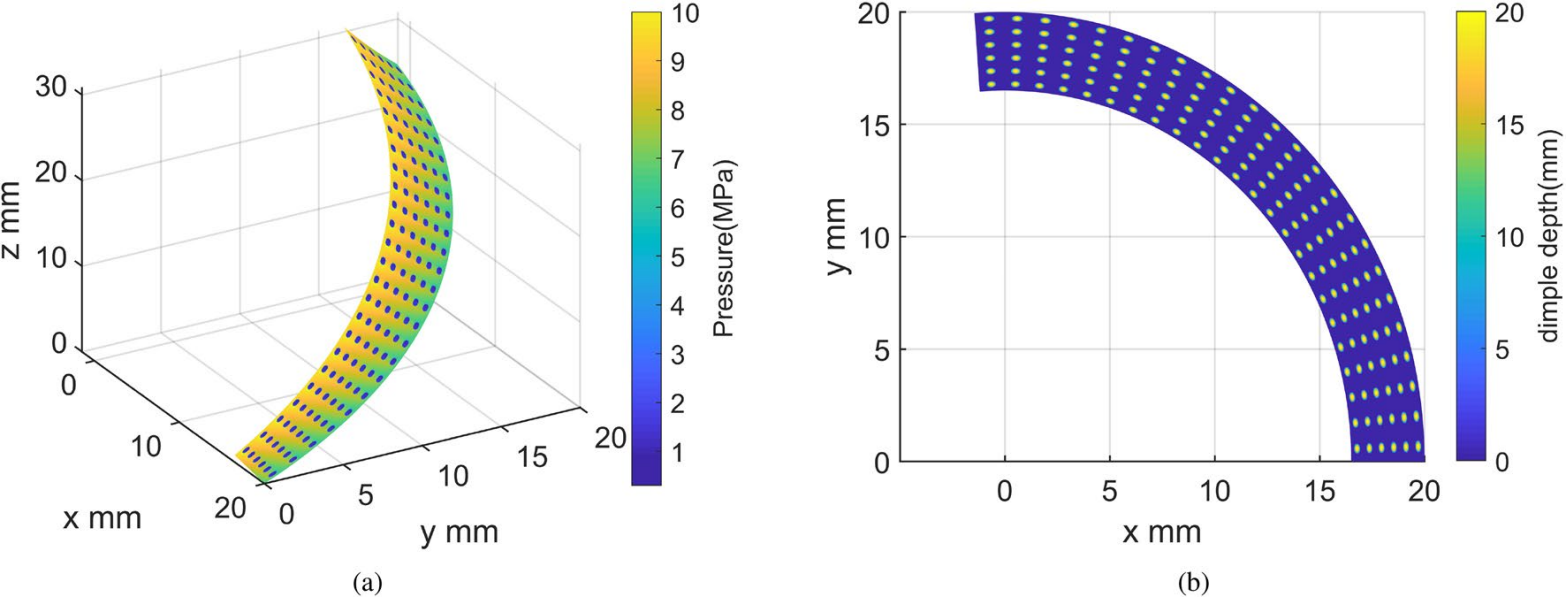
Initial design of surface texture. (**a**) Pressure distributions. (**b**) Top view of texture profile.

The optimal texture parameters are determined using the MEGO. The base design, built on experiential knowledge, acts as a reference group. The optimal design, reference group, smooth surface, and corresponding friction coefficients at various rotational speeds are showcased in Table 5. The percentage improvement in the friction coefficient after optimization is illustrated in Fig. 10. The pressure distributions and texture profiles of the optimal design and its reference group are depicted in Figs. 11 and 12, respectively.

**Table 5:** Final optimization results at different rotational speeds.

$\omega$/rpm	Design category	$r_{\textrm{t}}$	$Z_{\textrm{r}}$	$Z_{\textrm{c}}$	$\phi$/rad	$h_{\textrm{d}}$/um	$({\mu _{\textrm{cof}}})_{\min }$	Smooth$({\mu _{\textrm{cof}}})_{\min }$
15	Optimum	3	2	4	0	3.7	0.0759	0.0795
	Reference	1	6	24	0	20	0.0801	
30	Optimum	3	2	6	0	4.1	0.0702	0.0805
	Reference	1	6	24	0	20	0.0800	
60	Optimum	3	2	6	0	4.0	0.0598	0.0819
	Reference	1	6	24	0	20	0.0810	
90	Optimum	3	2	6	0	5.3	0.0518	0.0833
	Reference	1	6	24	0	20	0.0815	
120	Optimum	3	2	6	0	4.5	0.0439	0.0847
	Reference	1	6	24	0	20	0.0801	

As indicated in Fig. 11a, rapid shifts in pressure distribution can be observed in the vicinity of the dimples. The peak pressure is found at the starting position of the dimples along the sliding direction, a result of the additional hydrodynamic pressure from the converging wedge within this region. Conversely, a diverging wedge appears at the terminal position of the dimples along the sliding direction, inducing a negative pressure where cavitation transpires due to the Reynolds boundary conditions. Given the larger area of the positive pressure zone compared to the negative pressure zone and the influence of Reynolds boundary conditions, the textured area can provide an additional hydrodynamic load-bearing capacity. However, as shown in Fig. 12a, the additional hydrodynamic effects are not pronounced, and load-bearing is primarily facilitated by asperity contact. This leads to a reduction in film thickness and subsequently a gradient descent of pressure distribution along the radial direction across the entire surface, attributable to varying helix angles at the corresponding radius.

Table 5 demonstrates that all optimal texture parameters contribute positively to the reduction of the friction coefficient of the helical pair, with the extent of influence being associated with rotational speed. This can be attributed to the significant impact of sliding movement on the aforementioned additional hydrodynamic load-bearing capacity, as displayed in Fig. 11a. A certain range of high rotational speed can enhance this capacity. Moreover, the friction coefficient of the reference group exhibits negligible sensitivity to the rotational speed, implying scarce production of additional hydrodynamics within the reference group. At lower rotation speeds, such as 15 rpm, the friction coefficient can be curtailed by 4.5% using optimal texture parameters, while it experiences a 0.7% increase in the reference group. Although the impact on the friction coefficient is minimal in both cases, it reveals that the effectiveness of texture design in enhancing hydrodynamic lubrication is limited at very low rotation speeds. Consequently, both optimal and initially designed texture parameters yield similar outcomes, suggesting that the surface texture design of the helical pair could better serve as a secondary lubrication and wear particle accommodation mechanism rather than providing additional hydrodynamic lubrication.

At higher rotation speeds, the optimal texture design significantly curtails the friction coefficient, which stands at 0.0439 at 120 rpm. Compared to an untextured helical pair, the friction coefficient is reduced by 48.2% in the optimal texture design, while the reference group only achieves a 5.4% reduction. This translates to a 45.2% improvement in friction coefficient post-optimization, indicating that the friction coefficient is highly sensitive to texture parameters. A well-structured surface texture facilitates the generation of additional hydrodynamic lubrication at relatively high speeds.

Beyond 30 rpm, the optimal texture parameters remain almost unchanged and could be chosen as the optimal design for the helical pair, considering the limited influence of surface texture on friction coefficient at extremely low speeds. The optimal texture parameters are as follows: The number of radial dimples $Z_{\textrm{r}}$ is 2, the number of circumferential dimples $Z_{\textrm{c}}$ is 6, the dimple depth $h_{\textrm{d}}$ is 4-5.5 um, the axial ratio of the ellipse $r_{\textrm{t}}$ is 3, and the orientation of the major axis $\phi$ is 0$^{\circ }$. The geometric characteristics of the optimal texture parameters can be summarized as follows: a relatively small number of dimples in the radial and circumferential direction, implying larger dimple diameter and spacing, an appropriate dimple depth, a relatively flat ellipse shape, and an elliptical dimple shape with its major axis parallel to the sliding direction. These characteristics greatly enhance the hydrodynamic effect and significantly reduce the friction coefficient. The geometric characteristics of the optimal results align well with the study conducted by Yousfi et al.^41^, corroborated by experimental results. This is also evidenced by Fig. 11a, where compared to the reference group’s relatively large number of deep, circular dimples shown in Fig. 12a, the smaller number of shallow, elliptical dimples with a sliding direction parallel to the major axis creates a larger converging wedge, thus generating more additional hydrodynamic pressure.

### Effects of multiple parameters on friction coefficient

Given that a rotational speed of 120 rpm is a typical operating condition for the HHRA, and the most notable friction reduction effect is achieved at this speed following texture optimization, all subsequent parameter analyses will be conducted at 120 rpm. Moreover, the final values for the correlation parameter $\theta _{k}$ in the Kriging model, after iterations, are [0.0625, 0.0210, 4.0000, 2.6918, 0.7430].

**Figure 13:**
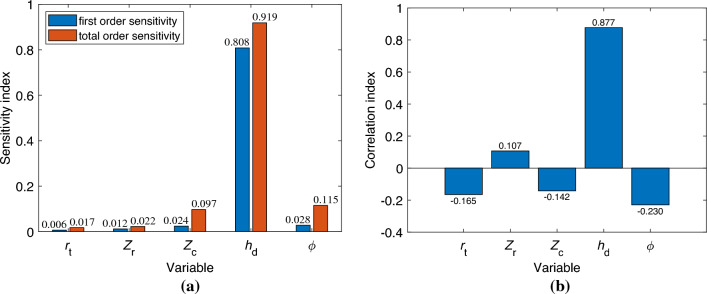
Analysis of sensitivity and correlation. (**a**) Sensitivity index. (**b**) Correlation index.

Understanding the impact of each design variable on the optimization target is crucial. To intuitively and quantitively estimate the influence of each parameter on the friction coefficient, a popular global sensitivity analysis method, Sobol’s method^42^, is utilized based on the built surrogate model. Figure 13a depicts the results where the bar lengths represent the total order sensitivity index and the first order sensitivity index, signifying the level of importance in affecting the friction coefficient. While the first order sensitivity index quantitively illustrates the significance of each variable, the total order sensitivity index conveys not just the effect of each variable on the target but also the interrelations among all design variables. As depicted in Fig. 13a, the first order sensitivity indices for the optimum axial ratio of the ellipse $r_{\textrm{t}}$, the number of radial dimples $Z_{\textrm{r}}$, the number of circumferential dimples $Z_{\textrm{c}}$, the dimple depth $h_{\textrm{d}}$, and the orientation of the major axis of the ellipse $\phi$ are 0.006, 0.012, 0.024, 0.808 and 0.028, respectively. Corresponding total order sensitivity indices are 0.017, 0.022, 0.097, 0.919, and 0.115. The markedly higher first order sensitivity indices of the dimple depth demonstrate its major contribution to the friction coefficient of the helical pair under these conditions. The negligible difference between the first order and total order sensitivity of $h_{\textrm{d}}$ suggests that, with constant area density, the effects of interactions between $h_{\textrm{d}}$ and other parameters on the friction coefficient are minimal and largely mutually independent, underscoring the pivotal role of dimple depth in lubricating film formation. With increased dimple depth, both the local additional hydrodynamic pressure and lubricating film thickness decrease, leading to enhanced micro-contact of asperities between the contact surfaces and subsequently, a rise in friction coefficient, and vice versa. Apart from dimple depth, the first order sensitivity indices of other parameters are quite marginal. However, the total order sensitivity indices of these parameters significantly surpass their first order counterparts, particularly for $Z_{\textrm{c}}$ and $\phi$, where total order sensitivity indices are approximately four times their first order sensitivity indices. This indicates that interactions among parameters, excluding dimple depth, have a greater influence on the friction coefficient than individual parameters. In essence, individually modulating parameters apart from dimple depth may not significantly alter the friction coefficient. However, adjusting these parameters in unison could considerably affect the friction coefficient. Therefore, it is crucial to judiciously match parameter relationships to positively impact friction reduction.

While the aforementioned sensitivity analysis can assist in understanding the degree to which each design variable of the texture structure impacts the friction coefficient, it does not reflect the relationship of the friction coefficient with variations in the design variables. In addition, the accuracy of the sensitivity analysis, which is based on a surrogate model, necessitates further verification. Therefore, this study employs the Spearman rank-order correlation^43^ to further investigate the relationship between the design variables and the objective function, and simultaneously verify the accuracy of the sensitivity analysis. Figure 13b exhibits the correlation coefficients of each design variable. It is found that the friction coefficient positively correlates with $Z_{\textrm{r}}$ and $h_{\textrm{d}}$, and negatively correlates with $r_{\textrm{t}}$, $Z_{\textrm{c}}$, and $\phi$. The correlation coefficient value of $h_{\textrm{d}}$ is greater than 0.8, indicating a strong positive correlation between the friction coefficient and the dimple depth. Consequently, a reasonable reduction in the dimple depth can effectively lower the minimum pressure. The absolute values of the correlation coefficients of the remaining design variables do not exceed 0.5. Therefore, it can be inferred that there is a lack of tight connection between the friction coefficient and the design variables, excluding $h_{\textrm{d}}$. This is consistent with the conclusion derived from the sensitivity analysis, further verifying its accuracy. The aforementioned correlation analysis and sensitivity analysis offer an effective method for exploring the internal relationships of the coupled parameters.

**Figure 14:**
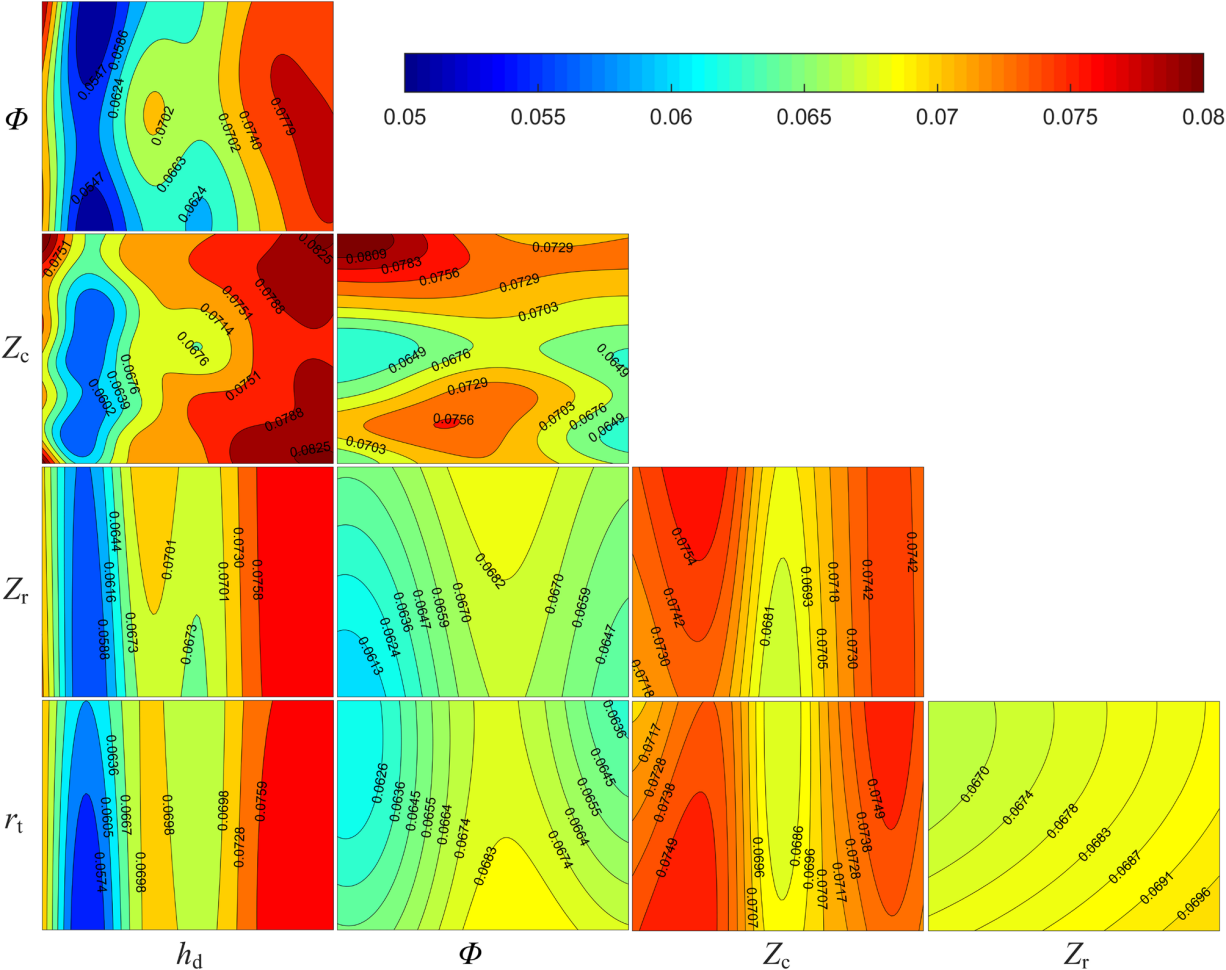
Multiple plots of friction coefficient with respect to design variables.

Figure 14 presents the contour plots of the friction coefficient, where each subplot reveals the influence of two design variables on the friction coefficient while the remaining variables are held at their median values, derived from the averages of the respective upper and lower boundaries. The contour plots related to $h_{\textrm{d}}$ exhibit the maximum friction coefficient difference of 0.0223, surpassing that of other design variables. This suggests that $h_{\textrm{d}}$ adjustments can significantly alter the friction coefficient, establishing its pronounced influence. Conversely, other design variables individually exert a lesser effect compared to $h_{\textrm{d}}$, particularly for $Z_{\textrm{c}}$ and $r_{\textrm{t}}$. This pattern aligns with the results in Fig. 13a. Moreover, in contour plots associated with $h_{\textrm{d}}$, the minimum friction coefficient value consistently appears near the first quintile of the $h_{\textrm{d}}$ range, approximately 4 mm. These parameters align with the optimal results from 5.2. The large gradient around this value shows that minor $h_{\textrm{d}}$ adjustments in this region can noticeably change the friction coefficient. In contrast, within the posterior two-thirds of the range, the small gradient signifies a lack of sensitivity of the friction coefficient to fluctuations in $h_{\textrm{d}}$. This indicates that with other design variables fixed, the friction coefficient markedly decreases to the trough and then slowly ascends with an increase in $h_{\textrm{d}}$. Additionally, in the $\phi -Z_{\textrm{c}}$ contour plot, the maximum friction coefficient difference is 0.0160, whereas the remaining plots unrelated to $h_{\textrm{d}}$ do not exceed 0.008. This suggests the significant influence of the combined effect of $\phi$ and $Z_{\textrm{c}}$ on the friction coefficient. The variables $\phi$ and $Z_{\textrm{c}}$ directly determine the circumferential distance between the dimples, significantly impacting the hydrodynamic effects. Increasing $Z_{\textrm{c}}$ and adjusting $\phi$ to favor the sliding direction reduces this distance, causing the additional hydrodynamic load capacity generated by each adjacent dimple to interact, thereby affecting the overall friction coefficient. In contrast, opposite adjustments to $Z_{\textrm{c}}$ and $\phi$ will increase the distance, reducing the interaction impact, which is directly reflected in the friction coefficient change. This principle is consistent with the results in Fig. 13a, where the total order sensitivity indices of $\phi$ and $Z_{\textrm{c}}$ are approximately four times their first order sensitivity indices.

**Figure 15:**
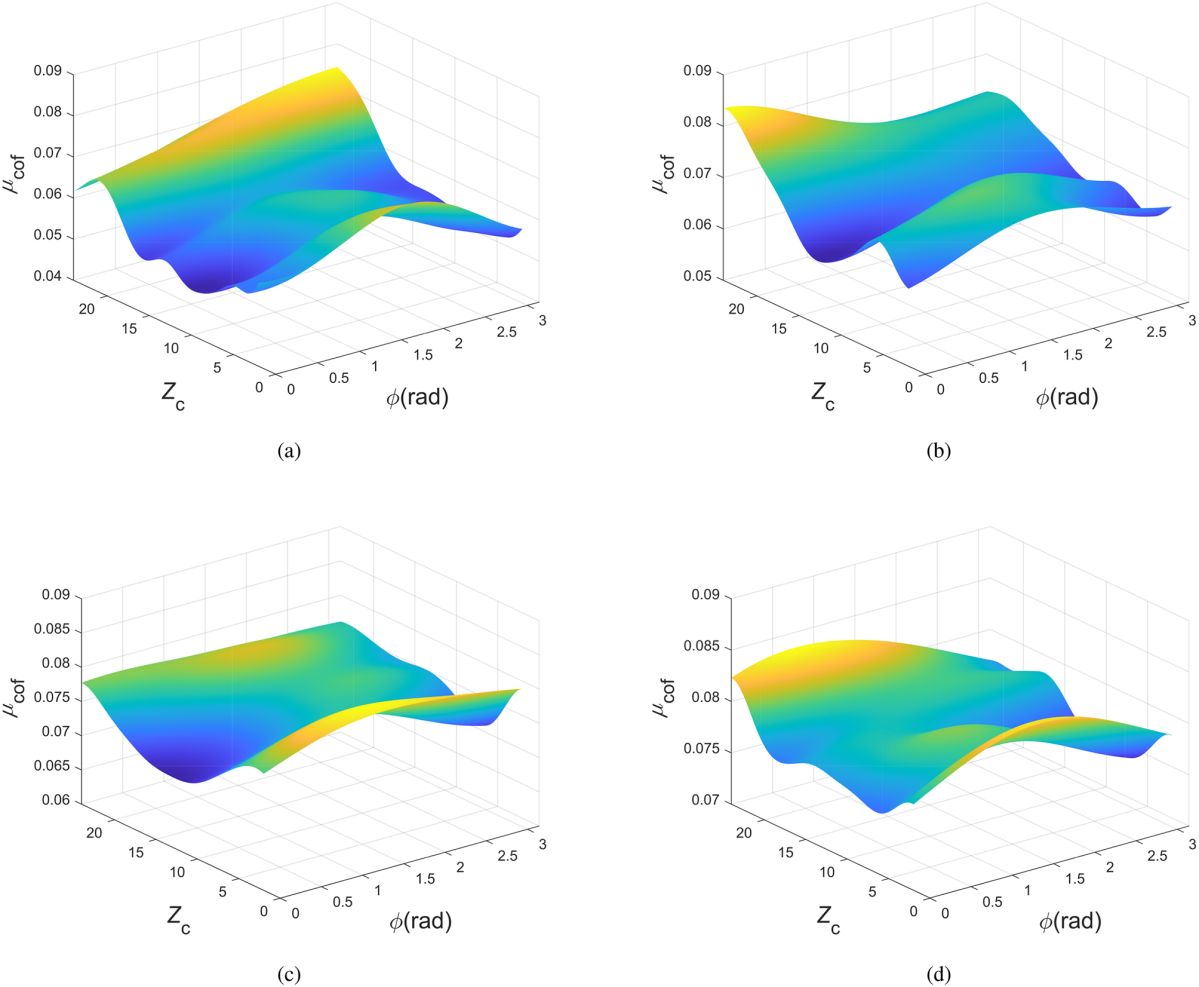
Relationship of ${\mu _{\textrm{cof}}}$ with variations in $Z_{\textrm{c}}$ and $\phi$ at different $h_{\textrm{d}}$. (**a**) $h_{\textrm{d}} = 5$ um (**b**) $h_{\textrm{d}} = 10$ um (**c**) $h_{\textrm{d}} = 15$ um (**d**) $h_{\textrm{d}} = 20$ um.

From the above analysis, it is apparent that the friction coefficient is mainly influenced by the independent effect of $h_{\textrm{d}}$, as well as the combined effect of $\phi$ and $Z_{\textrm{c}}$. To further explore whether the combined effect of $\phi$ and $Z_{\textrm{c}}$ on the friction coefficient changes with variations in $h_{\textrm{d}}$, other design variables are set to intermediate values. Figure 15 respectively illustrate the trend of friction coefficient changes with $\phi$ and $Z_{\textrm{c}}$ at different dimple depths $h_{\textrm{d}}$. It can be observed that all surfaces exhibit a “saddle shape”, i.e., they bulge at the sides and middle in the $Z_{\textrm{c}}$ direction and are concave at the sides and bulge in the middle along the $\phi$ direction. The consistency across all surfaces also suggests that the combined effect of $\phi$ and $Z_{\textrm{c}}$ is minimally influenced by $h_{\textrm{d}}$. Furthermore, within all surfaces, the friction coefficient generally escalates with the increase of $h_{\textrm{d}}$, which is in agreement with the previous analysis.

## Conclusion

This paper introduces a modified efficient global optimization method predicated on the Kriging model, devised to optimize the surface texture of the helical pair in HHRA. Initially, a surrogate model was constructed by integrating the lubrication model of the helical pair with MEGO. Subsequently, to affirm the efficacy of the proposed MEGO in surface texture design, a comparative analysis involving MEGO and multiple prevalent global optimization algorithms was undertaken. Ultimately, surrogate-based optimization and analysis were executed with the goal of minimizing the friction coefficient. Based on the analyses and results, the ensuing conclusions are made:

(1) Upon comparing various evaluation indices of the global optimization algorithms, the MEGO, in contrast to the EGO, Kriging + GA, BP + GA, and RBF +GA, demonstrates admirable proficiency in predicting the friction coefficient and optimizing texture parameters. This suggests that the MEGO can procure the optimal solution more rapidly while sustaining prediction accuracy.

(2) The dimple depth exerts the most substantial influence on the friction coefficient of the textured helical pair when the area density remains unchanged. Furthermore, the design parameters, excluding the dimple depth, interact mutually, exerting a more pronounced effect on the friction coefficient than any individual parameter, particularly in terms of the interaction between the orientation of the major axes of elliptical dimples and the number of circumferential dimples.

(3) Optimal texture parameters imply that large-sized, relatively flat elliptical texture features, with an appropriate dimple depth and the major axis oriented along the sliding direction, result in the most substantial reduction of the friction coefficient in the mixed lubrication regime.

(4) With the optimal texture parameters, the friction coefficient can be curtailed by as much as 45.2% compared with the initial design, underscoring the effectiveness of the proposed method in the surface texture optimization design process for superior tribological performance.

## Data Availability

The optimization process in this paper is based on the MATLAB. All necessary details are included in the paper and the other non-confidential solution files relevant to the present study can be obtained by contacting the corresponding author.

## References

[1] Zhang K (2022). Modeling and parameter sensitivity analysis of valve-controlled helical hydraulic rotary actuator system. Chin. J. Mech. Eng..

[2] Gachot C, Rosenkranz A, Hsu S, Costa H (2017). A critical assessment of surface texturing for friction and wear improvement. Wear.

[3] Wang W, He Y, Zhao J, Li Y, Luo J (2017). Numerical optimization of the groove texture bottom profile for thrust bearings. Tribol. Int..

[4] Zhang H (2017). Optimization of texture shape based on genetic algorithm under unidirectional sliding. Tribol. Int..

[5] Chen Y, Zhang J, Xu B, Chao Q, Liu G (2019). Multi-objective optimization of micron-scale surface textures for the cylinder/valve plate interface in axial piston pumps. Tribol. Int..

[6] Tang H, Ren Y, Xiang J, Anil K (2021). Evaluation and optimization of axial piston pump textured slipper bearings with spherical dimples based on hybrid genetic algorithm. Proc. Inst. Mech. Eng. Part J J. Eng. Tribol..

[7] Bei G, Ma C, Wang X, Sun J, Ni X (2022). On the optimal texture shape with the consideration of surface roughness. Sci. Rep..

[8] Hearst MA, Dumais ST, Osuna E, Platt J, Scholkopf B (1998). Support vector machines. IEEE Intell. Syst. Appl..

[9] Matheron G (1963). Principles of geostatistics. Econ. Geol..

[10] Santner, T. J., Williams, B. J., Notz, W. I. & Williams, B. J. *The design and analysis of computer experiments*, vol. 1 (Springer, 2003).

[11] Hardy RL (1971). Multiquadric equations of topography and other irregular surfaces. J. Geophys. Res..

[12] Kostić S, Vasović N, Marinković B (2017). Robust optimization of concrete strength estimation using response surface methodology and monte carlo simulation. Eng. Optim..

[13] Tang T (2020). A hydrodynamic prediction model of throttle orifice plate using space filling and adaptive sampling method. Struct. Multidiscip. Optim..

[14] Guzman Nieto, M., ElSayed, M. S. & Walch, D. Efficient global optimization and modal strain energy coefficient-based algorithm for fast prediction of dynamic aeroelastic loads. *Struct. Multidiscip. Optim.* **60**, 817–834 (2019).

[15] Jing Z, Chen J, Li X (2019). Rbf-ga: An adaptive radial basis function metamodeling with genetic algorithm for structural reliability analysis. Reliab. Eng. Syst. Saf..

[16] Gao Z (2016). Parameters optimization of hybrid fiber laser-arc butt welding on 316l stainless steel using kriging model and ga. Opt. Laser Technol..

[17] Chen, W., Wang, P. & Dong, H. Surrogate-based bilevel shape optimization for blended-wing–body underwater gliders. *Eng. Optim.* 1–22 (2022).

[18] Jones DR, Schonlau M, Welch WJ (1998). Efficient global optimization of expensive black-box functions. J. Global Optim..

[19] Jeong S, Murayama M, Yamamoto K (2005). Efficient optimization design method using kriging model. J. Aircr..

[20] Ghoreyshi M, Badcock K, Woodgate M (2009). Accelerating the numerical generation of aerodynamic models for flight simulation. J. Aircr..

[21] Ariyarit A, Sugiura M, Tanabe Y, Kanazaki M (2018). Hybrid surrogate-model-based multi-fidelity efficient global optimization applied to helicopter blade design. Eng. Optim..

[22] Ponweiser, W., Wagner, T. & Vincze, M. Clustered multiple generalized expected improvement: A novel infill sampling criterion for surrogate models. In *2008 IEEE congress on evolutionary computation (IEEE World Congress on Computational Intelligence)*, 3515–3522 (2008).

[23] McKay M, Beckman R, Conover W (1979). Acomparisonof three methodsforselecting valuesofinputvariablesinthe analysisofoutputfrom acomputercode. Technometrics.

[24] Stein M (1987). Large sample properties of simulations using latin hypercube sampling. Technometrics.

[25] Liefvendahl M, Stocki R (2006). A study on algorithms for optimization of latin hypercubes. J. Stat. Plan. Inference.

[26] Sacks J, Welch WJ, Mitchell TJ, Wynn HP (1989). Design and analysis of computer experiments. Stat. Sci..

[27] Sóbester A, Leary SJ, Keane AJ (2005). On the design of optimization strategies based on global response surface approximation models. J. Global Optim..

[28] Yin X, Goudriaan J, Lantinga EA, Vos J, Spiertz HJ (2003). A flexible sigmoid function of determinate growth. Ann. Bot..

[29] El-Sayed H, Khatan H (1974). The exact performance of externally pressurized power screws. Wear.

[30] Yu H, Wang X, Zhou F (2010). Geometric shape effects of surface texture on the generation of hydrodynamic pressure between conformal contacting surfaces. Tribol. Lett..

[31] Dobrica M, Fillon M (2009). About the validity of reynolds equation and inertia effects in textured sliders of infinite width. Proc. Inst. Mech. Eng. Part J J. Eng. Tribol..

[32] Zhang Y, Lu C, Liu Y (2018). Averaging effect on pitch errors in hydrostatic lead screws considering helical recess layout and nut misalignment. Proc. Inst. Mech. Eng. Part J J. Eng. Tribol..

[33] Greenwood JA, Tripp J (1970). The contact of two nominally flat rough surfaces. Proc. Inst. Mech. Eng..

[34] Patir, N. & Cheng, H. S. An average flow model for determining effects of three-dimensional roughness on partial hydrodynamic lubrication. *Trans. Asme J. Lubr. Technol.* **100**, 12–17 (1978).

[35] Patir N, Cheng HS (1979). Application of average flow model to lubrication between rough sliding surfaces. J. Lubr. Technol..

[36] Wu C, Zheng L (1989). An average reynolds equation for partial film lubrication with a contact factor. J. Tribol..

[37] Kango S, Singh D, Sharma R (2012). Numerical investigation on the influence of surface texture on the performance of hydrodynamic journal bearing. Meccanica.

[38] Xu Y (2019). Influence of dimple shape on tribofilm formation and tribological properties of textured surfaces under full and starved lubrication. Tribol. Int..

[39] Nuchitprasittichai A, Cremaschi S (2013). An algorithm to determine sample sizes for optimization with artificial neural networks. AIChE J..

[40] Yin F, Mao H, Hua L (2011). A hybrid of back propagation neural network and genetic algorithm for optimization of injection molding process parameters. Mater. Des..

[41] Yousfi M, Mezghani S, Demirci I, El Mansori M (2016). Tribological performances of elliptic and circular texture patterns produced by innovative honing process. Tribol. Int..

[42] Sobol IM (2001). Global sensitivity indices for nonlinear mathematical models and their monte carlo estimates. Math. Comput. Simul..

[43] Prion S, Haerling KA (2014). Making sense of methods and measurement: Spearman-rho ranked-order correlation coefficient. Clin. Simul. Nurs..

